# Biogeochemical water type influences community composition, species richness, and biomass in megadiverse Amazonian fish assemblages

**DOI:** 10.1038/s41598-020-72349-0

**Published:** 2020-09-18

**Authors:** Juan David Bogotá-Gregory, Flávio C. T. Lima, Sandra B. Correa, Cárlison Silva-Oliveira, David G. Jenkins, Frank R. Ribeiro, Nathan R. Lovejoy, Roberto E. Reis, William G. R. Crampton

**Affiliations:** 1 Department of Biology, University of Central Florida, 4100 Libra Dr, Orlando, FL 32816 USA; 2 Museu de Zoologia da Universidade Estadual de Campinas, Cidade Universitária, Campinas, São Paulo 13083-863 Brazil; 3 Department of Wildlife, Fisheries and Aquaculture, Mississippi State University, Box 9680, Starkville, MS 39762 USA; 4 Instituto Nacional de Pesquisas da Amazonia, Coleção de Peixes, Av. André Araújo, 2936, Petrópolis, Manaus, Amazonas 69.067-375 Brazil; 5 Coleção Ictiológica da Universidade Federal do Oeste do Pará. Campus Amazônia, Avenida Mendonça Furtado, 2946, Fátima, Santarém, Pará CEP 68040-470 Brazil; 6 Department of Biological Sciences, University of Toronto Scarborough, 1265 Military Trail, Toronto, ON M1C 1A4 Canada; 7 Pontifícia Universidade Católica do Rio Grande do Sul, Avenida Ipiranga 6681, Porto Alegre, Rio Grande do Sul 90619-900 Brazil

**Keywords:** Ecology, Community ecology, Freshwater ecology, Tropical ecology, Wetlands ecology

## Abstract

Amazonian waters are classified into three biogeochemical categories by dissolved nutrient content, sediment type, transparency, and acidity—all important predictors of autochthonous and allochthonous primary production (PP): (1) nutrient-poor, low-sediment, high-transparency, humic-stained, acidic *blackwaters*; (2) nutrient-poor, low-sediment, high-transparency, neutral *clearwaters*; (3) nutrient-rich, low-transparency, alluvial sediment-laden, neutral *whitewaters*. The classification, first proposed by Alfred Russel Wallace in 1853, is well supported but its effects on fish are poorly understood. To investigate how Amazonian fish community composition and species richness are influenced by water type, we conducted quantitative year-round sampling of floodplain lake and river-margin habitats at a locality where all three water types co-occur. We sampled 22,398 fish from 310 species. Community composition was influenced more by water type than habitat. Whitewater communities were distinct from those of blackwaters and clearwaters, with community structure correlated strongly to conductivity and turbidity. Mean per-sampling event species richness and biomass were significantly higher in nutrient-rich whitewater floodplain lakes than in oligotrophic blackwater and clearwater river-floodplain systems and light-limited whitewater rivers. Our study provides novel insights into the influences of biogeochemical water type and ecosystem productivity on Earth’s most diverse aquatic vertebrate fauna and highlights the importance of including multiple water types in conservation planning.

## Introduction

The Amazon basin contains the most diverse riverine vertebrate assemblage on Earth, with ca. 2,800 species (7.8% of planetary fish diversity^1^) distributed across multiple sub-basins and aquatic habitat types^2–4^. Efforts to understand the diversity and distribution of Amazonian fish have focused on paleogeographic and paleoclimatic events^5^, species-area effects^6,7^, river continuum effects^7,8^, habitat specialization^2,9^, ecomorphological, physiological, and sensory specializations^10–12^, biotic interactions^13^, and neutral processes^14^. Nonetheless, our understanding of how fish assemblages are structured by variation in basic physico-chemical water properties is surprisingly incomplete.

**Figure 1:**
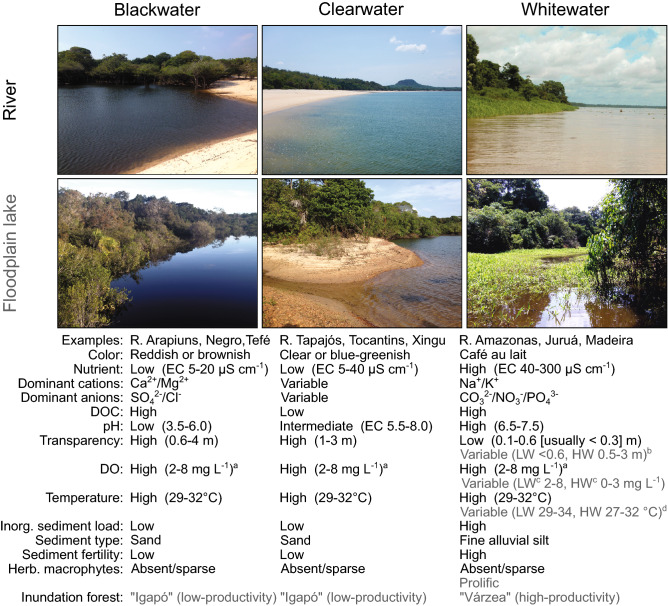
The Wallace classification of Amazonian rivers, with typical ranges of physico-chemical properties. Values for floodplain lakes (gray text) are given only where they typically differ from parent rivers. Ranges follow Junk et al.^17^, Crampton^2^ and Appendix S4 online. *EC* conductivity, *DOC* dissolved organic carbon, *LW/HW* low/high water, *DO* dissolved oxygen, *Inorg.* Inorganic, *Herb.* herbaceous. ^a^Periodic phytoplankton (including cyanobacteria) blooms induce DO supersaturation (ca. 8–15 mg L^−1^) and tint clearwater green. ^b^Precipitation of suspended silt due to reduced flow in WW floodplain lakes substantially increases transparency relative to parent WW river. ^c^HW hypoxia results from litter decomposition in inundation forests; this effect is greater in large WW floodplains. ^d^Shallow WW lakes reach extreme high LW temperature. Photographs are of the R. Arapiuns (blackwater), Tapajós (clearwater) and Amazonas (whitewater) near Santarém, Brazil.

Amazonian waters are classified into three distinct biogeochemical categories (Fig. 1) by a combination of dissolved nutrient content, sediment type, transparency, and acidity—all important determinants of autochthonous and allochthonous primary productivity (PP): (1) nutrient-poor, low-sediment, high-transparency (but humic-stained), and acidic *blackwaters* originating from deeply weathered soils overlying lowland tropical forests and savannas of the Amazon’s intracratonic basin or shield fringes^15–19^; (2) nutrient-poor, low-sediment, high-transparency, and neutral *clearwaters* originating from upland shield formations dominated by non-biogenous Precambrian rocks^15,17–19^; (3) nutrient-rich, low-transparency, neutral *whitewaters*, with high loads of fertile alluvial suspended sediment. Whitewaters originate from contemporary or historical Andean headwaters^15,17–19^ and derive their high sediment load and high concentration of dissolved nutrients (that is, the dissolved ions that limit autotrophic production, e.g. calcium, magnesium, nitrate, phosphate) from the erosion of biogenous sediments of marine provenance^20–24^. In contrast, the highly weathered soils and dense vegetation of Amazonian blackwaters and clearwater drainages lead to low sediment load and low dissolved nutrient content^16,17,19,21,22^ (conductivity ca. 5–40 μS cm^−1^, versus ca. 40–300 [usually > 60] μS cm^−1^ for whitewaters).

The three water types were first described by Alfred Russel Wallace^25^ in one of the earliest scientific accounts of Amazônia and their biogeochemical bases were later elucidated by geochemical and limnological studies^18,21,26,27^. Wallace’s classification remains in use and is congruent with modern river classifications based on synthetic aperture radar imaging^28^. Nonetheless, the effects of biogeochemical water type on Amazonian fish assemblages remain poorly documented. For instance, our understanding of disparities in fish assemblage structure between water types is mostly based on comparisons of sites separated by hundreds or thousands of kilometers, where the influence of water type is confounded by geographical variation in species distributions. In contrast, few studies have compared assemblage structure between water types located in close geographical proximity. These include comparisons of black- versus whitewater by Galacatos et al.^29^, Henderson and Crampton^30^, and Saint-Paul et al.^31^, and black- versus clearwater by Winemiller et al.^32^; these studies described distinctive communities of species associated with each water type. However, a simultaneous comparison of the fish faunas of adjacent or nearby black-, clear- and whitewater systems has yet to be undertaken to unravel how biogeochemical disparities influence community composition.

The effects of dissolved nutrient content, sediment type, transparency, and acidity on the autochthonous and allochthonous PP of Amazonian aquatic habitats have been well studied. Three main patterns emerge: (1) Phytoplankton and algal periphyton PP is high in sediment-decanted nutrient-rich whitewater floodplain lakes^27,33–39^, lower in oligotrophic (nutrient-limited) blackwater and clearwater rivers and floodplains^16,21,27,34,35,39–41^ (especially low in blackwaters with extreme low pH^42^), and negligible in whitewater river channels, where photosynthesis is light-limited by the high suspended sediment load^20,27,43,44^. Second, extensive stands of grass-dominated herbaceous macrophytes^45–48^ and associated algal epiphyton^49^ develop in whitewater lakes. These grasses have a free-floating high-water stage dependent on the high dissolved nutrient content of whitewaters, but also a rooted low-water stage dependent on the high fertility of the alluvial deposits of whitewaters^47^. In contrast herbaceous macrophytes are generally absent (or very sparse) in black- and clearwater floodplains due to the low dissolved nutrient content and infertile sand-based lake sediments^22,50^. Herbaceous macrophytes growth is also minimal in whitewater rivers (despite high nutrient availability) due to the strong currents. Third, high-productivity ‘várzea’ inundation forests and grasslands, with associated high allochthonous inputs (e.g. seeds and arthropods^51^), develop on the fertile alluvial floodplain deposits surrounding whitewater floodplain lakes^17,51–57^. In contrast low-productivity, often stunted, ‘igapó’ inundation forests develop on clear- and blackwater floodplains^17,52–57^.

Species-energy relationships predict higher fish diversity in systems with higher total PP^58,59^, which would suggest that whitewater floodplains (but not necessarily light-limited whitewater rivers) might support higher species richness than blackwater and clearwater rivers and floodplains. Our current understanding of aquatic species-energy relationships in the Amazon has been greatly influenced by Goulding et al.’s^16^ book ‘Rio Negro: Rich life in poor water’, which introduced the notion that highly oligotrophic waters can support paradoxically high fish species diversity – mirroring patterns in coral reefs^60^. The authors proposed that high diversity can occurs despite low autochthonous PP because the energy sustaining fish communities derives primarily from allochthonous inputs from seasonally flooded forests. Nonetheless, only two quantitative comparisons of fish species richness between Amazon water types are available. Saint-Paul et al.^31^ described higher diversity in the blackwater R. Negro than the mainstem whitewater R. Amazonas, but Henderson and Crampton^30^ reported a contrary pattern of higher diversity in the R. Amazonas than the blackwater R. Tefé.

**Figure 2:**
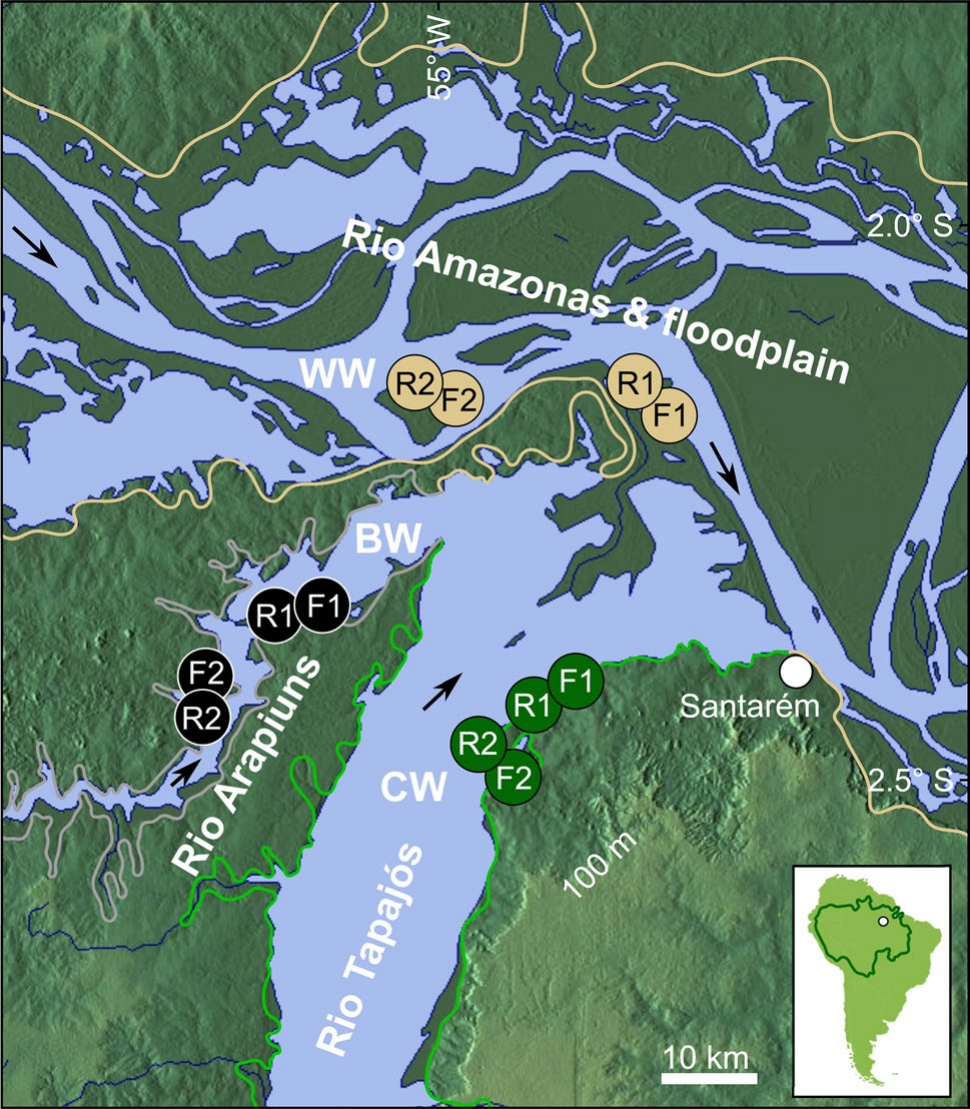
Map of the study area near Santarém, Brazil, with locations of the three sampled *water types* (blackwater *BW*, clearwater *CW*, whitewater *WW*) and two sampled *habitat types* (river margin *R*, floodplain lake *F*). Solid lines represent boundaries of seasonal floodplains. Arrows indicate river flow. Dark green line in inset map of South America demarcates Amazon river drainage. Sampling was conducted through a full annual hydrological cycle, every two months, at two *site replicates* (labeled 1 and 2) representing each of the six combinations of water type and habitat type. This yielded a planned total of 72 sample events, one of which was missed due to bad weather, leaving 71. Base map from MFF-maps (https://maps-for-free.com) modified with Inkscape version 0.92 (www.inkscape.org).

To assess the influence of water type on Amazonian fishes we conducted a year-round quantitative sampling program of river margin and floodplain habitats in one of the few areas of Amazônia where all three biogeochemical water types occur in close proximity (near Santarém, Brazil, Fig. 2). Based on the results of this sampling program we discuss the role of water type and habitat in shaping fish assemblage structure, species richness, and biomass. Our analyses also address whether habitats known to exhibit both high autochthonous and allochthonous PP (i.e. whitewater lakes) exhibit higher species richness (and/or biomass) than those known to exhibit low autochthonous PP and relatively reduced allochthonous inputs (i.e. floodplain lakes and main river channels of blackwater and clearwater systems, and nutrient-rich but light-limited whitewater river channels).

## Results

### Physico-chemical parameters

**Figure 3:**
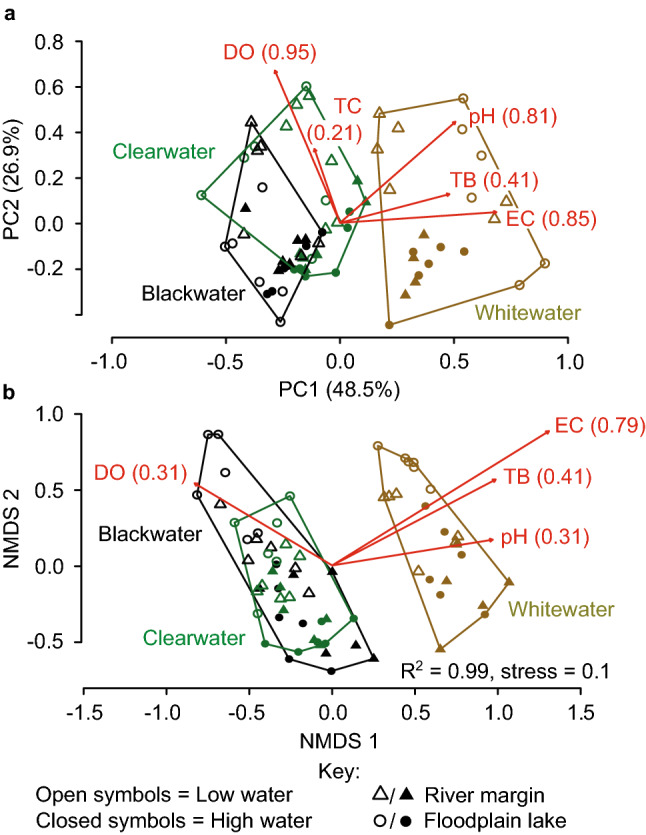
Multivariate analyses of physico-chemical water properties and fish assemblage structure in the study area. (**a**) Principal component analysis of physico-chemical properties measured at 71 sampling events through a complete annual hydrological cycle. (**b**) Nonmetric multidimensional scaling ordination based on fish assemblage structure at the same 71 sampling events. Red arrows represent environmental vectors significant at α = 0.05, with R^2^ values. *DO* dissolved oxygen, *EC* conductivity, *TB* turbidity, *TC* temperature.

Our measurements of physico-chemical parameters in the blackwater (BW) R. Arapiuns, clearwater (CW) R. Tapajós, and whitewater (WW) R. Amazonas (Supplementary Table S1 online) were within the typical ranges summarized in Fig. 1 for BW, CW and WW rivers. All pairwise combinations of the three water types differed significantly in physico-chemical properties; permutational multivariate analysis of variance (PERMANOVA): BW-CW, (pseudo) *F* = 7.2, *R* = 0.73, *P* < 0.001; BW-WW, *F* = 67.9, *R* = 0.77 *P* < 0.001; CW-WW, *F* = 42.0, *R* = 0.69, *P* < 0.001; global, *F* = 38.4, *R* = 0.73, *P* < 0.001 (Supplementary Table S2 online for differences subdivided by habitat type, i.e. floodplain lake, river margin, all were significant at α = 0.001). In a principal components analysis (PCA) ordination (Fig. 3a), sampling events formed two non-overlapping groups—one represented by BW and CW, and another by WW. Sampling events for BW and CW overlapped at low water and high water. We observed no segregation of the BW and CW sampling events at 3rd or higher PCA axes. The environmental vectors for turbidity, conductivity, and pH increased in the order BW–CW–WW.

### Habitat structure, vegetation, and area

Substrate and vegetation types were typical for Amazon black-, clear-, and whitewater rivers-floodplain systems (Fig. 1). Herbaceous macrophytes were restricted to the whitewater floodplain and comprised dense stands of *Echinochloa polystachya* and other grasses with a marginal rooted low-water stage and floating high-water stage, as well as some other macrophytes, e.g. *Eichhornia* spp. In a rectangular cross-river transect of 10 km width at the R2 sites (including both banks and intervening islands), we measured the WW floodplain area as ca. 140 km^2^ per 10 km river length, 35–47 times the area of BW (4 km^2^) and CW floodplains (3 km^2^), respectively.

### Fish community composition

We collected 22,398 fish from 310 species, 172 genera, 44 families, and 15 orders (Supplementary Tables S3, Appendix S1 online). 89% of species were identified as valid described species, 6% as undescribed species (listed ‘sp.’), and 5% were assigned ‘conferre’ (cf.) to described species in need of taxonomic revision.

#### Species richness

**Figure 4:**
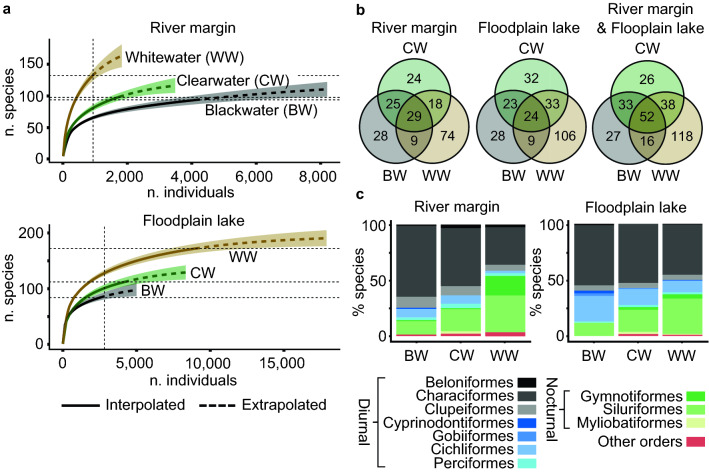
Patterns of fish species richness and taxonomic composition among the three water types and two habitat types of the study area. (**a**) Rarefaction curves; see Supplementary Figs. S4 and S5 online for similar patterns in rarefaction curves divided by site replicate and by gear type, respectively. (**b**) Species occupancy Venn diagrams per habitat type, reporting number of exclusive and shared species; see Supplementary Fig. S6 online for similar patterns in Venn Diagrams restricted to single site replicates. (**c**) Habitat specialization (% species occurrences per habitat type); Other Orders are Osteoglossiformes, Tetraodontiformes, Pleuronectiformes, Synbranchiformes, and Ceratodontiformes. Orders are assigned to predominantly diurnal or nocturnal activity following the literature cited in Supplementary Appendix S5 online.

WW habitats exhibited higher total species richness than BW and CW habitats (Table 1, Fig. 4a). With the removal of rare species (those with < 4 individuals), WW floodplain still exhibited the highest species richness, but river habitats exhibited similar richness (tied in WW and BW, slightly lower in CW, Table 1). The percentage of rare species in WW rivers greatly exceeded that of other water/habitat type combinations (Table 1). Rarefaction curves (Fig. 4a) reached asymptotes for all water type/habitat combinations, except for WW rivers for which species richness may be underestimated (but nonetheless exceeded that of CW and BW rivers). Rarified species richness estimates were invariably highest in WW and mostly lowest in BW, both for floodplain and river sites, despite low abundance in WW rivers (Table 1).

**Table 1:** Summary results for fish species richness, biomass, and abundance in the study area, including common indices of richness, effective species number, diversity, and evenness. Means refer to mean per sample event.

Habitat type	Water type	Total species richness	Mean species richness	Species richness minus rare species	% rare	Rarefied species richness	Chao1 estimated species richness	Jost diversity	Simpson diversity	Pielou evenness	Total Biomass (kg)	Mean Biomass (kg)	Total Abundance (n fish)	Mean abundance (n fish)
River margin	Blackwater	91	**20.6**	***75***	21.3	62.2	122.6	17.7	0.88	0.64	**45.8**	**3.81**	**4,098**	**223.3**
	Clearwater	96	17.5	71	35.2	77.7	121.4	21.4	0.88	0.67	31.3	2.61	1,739	144.9
	Whitewater	**130**	19.5	***75***	**73.3**	**130.0**	**181.3**	**53.6**	**0.97**	**0.82**	35.0	3.17	911	38.2
	Total	207									112.0		6,748	
Floodplain lake	Blackwater	84	16.3	61	37.7	84.0	105.1	25.6	0.93	**0.73**	28.0	1.97	2,519	209.9
	Clearwater	112	21.2	87	28.7	97.8	135.6	20.4	0.90	0.64	54.2	2.36	4,166	97.4
	Whitewater	**172**	**43.9**	**117**	**47.0**	**123.9**	**194.9**	**33.8**	**0.94**	0.68	**136.5**	**11.38**	**8,965**	**533.8**
	Total	255									218.8		15,650	
River margin & Floodplain lake	Blackwater	128	28.75	105	21.9	124.6	186.1	30.1	0.93	**0.70**	73.8	4.80	6,617	**328.3**
	Clearwater	149	28.10	122	22.1	149.0	190.0	29.3	0.93	0.68	85.6	3.79	5,905	193.6
	Whitewater	**224**	**41.45**	**163**	**37.4**	**199.6**	**253.6**	**44.1**	**0.95**	0.70	**171.50**	**8.86**	**9,876**	305.1
	Total	310									330.8		22,398	

The highest values for each habitat type are reported in bold for emphasis (tied highest values in bold and italics).

#### Biomass and abundance

Total biomass and abundance in floodplains substantially exceeded that of rivers (Table 1). For floodplains, both total biomass and total abundance were higher in WW than in BW and CW. However, in rivers, the highest biomass and abundance was observed in BW. The total abundance of river fish was largely decoupled from total biomass due to variation in average fish weight: in BWs (0.011 kg), CW (0.017 kg), and WW (0.038 kg).

#### Assemblage structure

The proportion of species exclusive to WW exceeded that of BW and CW. This pattern occurred in rivers, floodplains, and both habitats pooled (Fig. 4b). In both river and floodplain habitats, the predominantly diurnally-active Characiformes, Clupeiformes, Cichliformes, and Perciformes decreased in species richness in the order BW–CW–WW, while the nocturnally active Siluriformes and Gymnotiformes exhibited an opposite pattern (Fig. 4c). Siluriformes and Gymnotiformes were especially abundant in the whitewater river samples.

Assemblage structure differed significantly for all pairwise combinations of water type by PERMANOVA, with BW and CW exhibiting the greatest similarity (lowest *R*); BW-CW, (pseudo) *F* = 3.8, *R* = 0.27, *P* < 0.001; BW-WW, *F* = 8.5, *R* = 0.4, *P* < 0.001; CW-WW, *F* = 42.0, *R* = 9.8, *P* < 0.001; global, *F* = 7.4, *R* = 0.41, *P* < 0.001. Supplementary Table S4 online reports disparities subdivided by habitat type (all significant at α = 0.001).

About half the variation in assemblage structure was attributable to variation in physico-chemical parameters; co-inertia analysis (COIA) RV value = 0.46, *P* < 0.001, null model 95% CIs 0.125–0.134. In an analysis presented in Supplementary Appendix S2 online we confirmed that the relationship between assemblage structure and physico-chemical parameters had negligible spatial dependence (was unaffected by hydrological distances among pairs of sites).

Like in the PCA of physico-chemical properties (Fig. 3a), an ordination of assemblage structure by nonmetric multidimensional scaling (NMDS) (Fig. 3b) revealed two non-overlapping groups: one corresponding to WW habitats, and another to BW and CW habitats. For all three water types, floodplain and river sampling events overlapped considerably in the NMDS ordination of assemblage structure, indicating that community composition is more strongly affected by water type than by habitat structure (Fig. 3b). The environmental vectors for turbidity, conductivity, and pH loaded positively in a direction from the BW + CW group to the WW group, with conductivity and turbidity exhibiting the strongest correlation with assemblage structure. Similar outcomes occurred if rare species were removed (Supplementary Fig. S1, online), demonstrating that rare species have little influence on variation in assemblage structure. Similar outcomes also occurred in separate ordinations for the two utilized gear types (gill nets and seine nets) (Supplementary Fig. S2, online)—confirming that our analyses of community composition were not biased by gear type, as well as in separate ordinations for site replicates 1 and 2 (Supplementary Fig. S3, online)—confirming that none of our site replicate pairs had major discrepancies in species composition.

**Figure 5:**
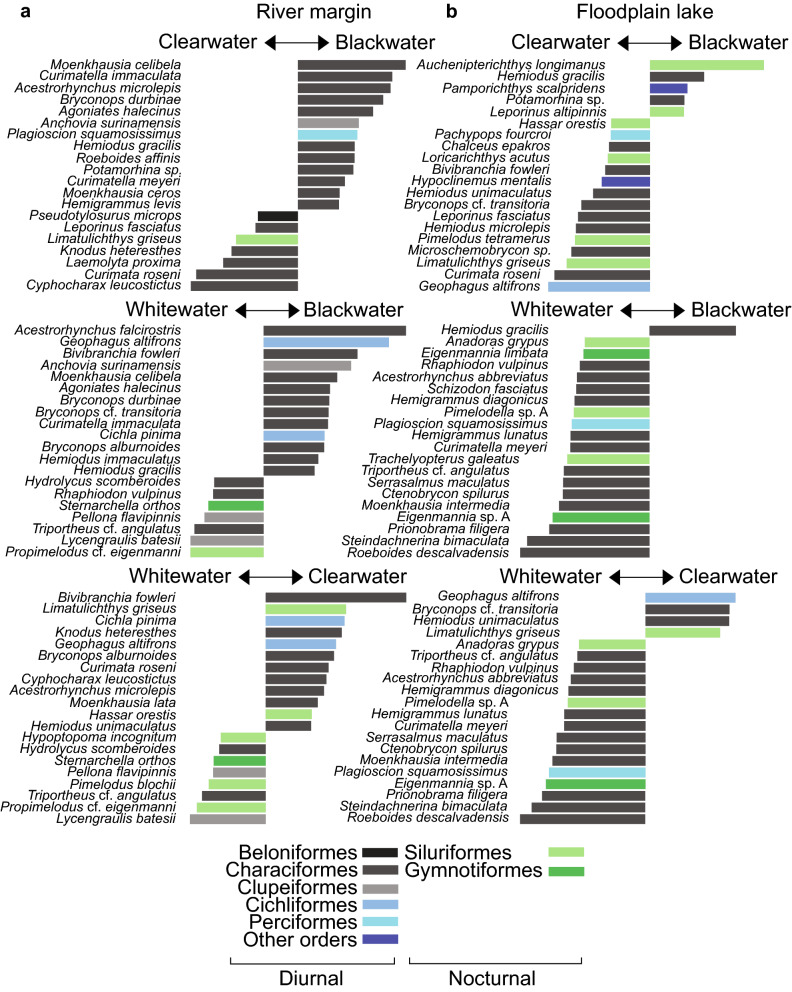
Contributions of the 20 most influential species to the differences in assemblage structure between water types, based on PERMANOVA coefficients. Length of bars represent the magnitude of contribution to difference between each pairwise combination of water type.

Species diagnostic of WW (especially rivers) comprised a mixture of diurnally active groups and nocturnally active Siluriformes and Gymnotiformes (Fig. 5). In contrast, species exerting the strongest influence on differences in assemblage structure between BW and CW belonged to the predominantly diurnal orders Clupeiformes, Characiformes, and Cichliformes.

### Quantitative comparisons of species richness, biomass, and abundance

**Figure 6:**
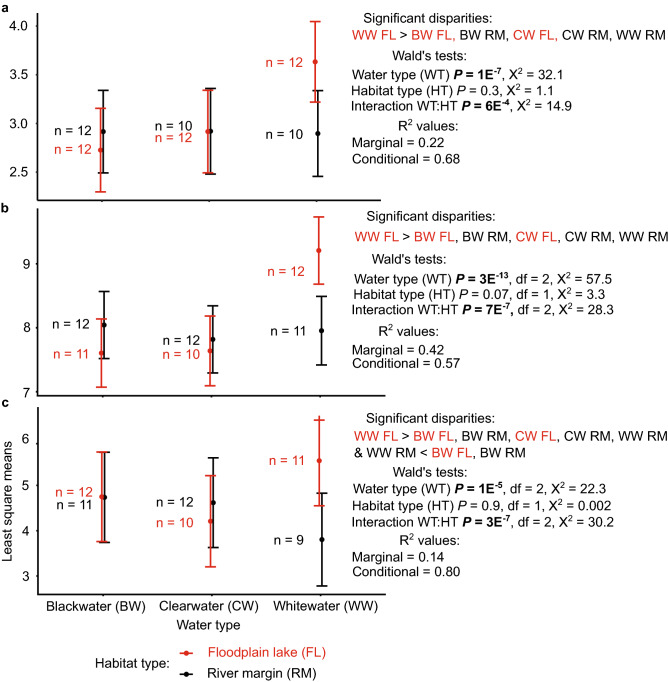
Generalized linear mixed-effect models for the effects of water type and habitat type on: (**a**) species richness, (**b**) biomass, and (**c**) abundance. Plots show least square means for the fixed effects (water type, habitat) with 95% confidence intervals. Disparities are reported where significant at α = 0.05 by Tukey post-hoc test (see Supplementary Table S5 online for model details). Sample sizes (n) refer to sampling events. Here outlier sampling events (reported in Supplementary Fig. S7 online) were excluded, hence variation in sample sizes between models.

Generalized linear mixed-effect models (GLMMs) showed that WW floodplain lakes significantly exceeded all other water/habitat type combinations for mean (per-sampling event) species richness (Fig. 6a), biomass (Fig. 6b), and abundance (Fig. 6c). *Species richness:* Mean WW floodplain species richness exceeded that of BW and CW floodplains 2.7-fold and 2.1-fold, respectively, and exceeded that of all river margin sites (2.1–2.5-fold). In river margins, we observed no significant differences in species richness among water types, although the highest values were reported for BW. *Biomass:* Mean WW floodplain biomass exceeded that of BW and CW floodplains 4.9-fold and 2.5-fold, respectively, and exceeded that of all river margin sites (3.0–4.3-fold) (Table 1). *Abundance:* Finally, mean WW floodplain abundance exceeded that of BW and CW floodplains 2.5-fold and 5.4-fold, respectively, and exceeded that of all river margin sites (2.3–14.0-fold). Mean BW river abundance exceeded that of WW rivers 5.8-fold (although this disparity was lost in most of the repeat analyses reported in Table S3), but abundance in CW did not differ significantly from BW or WW. Due to lower average fish weight in BW than WW river habitats (see above), the difference in mean abundance between BW and WW rivers (5.8-fold) was larger than the difference in mean biomass (1.3-fold).

The significant differences reported in Fig. 6 are robust because they were also recovered in: (1) GLMMs for richness, biomass, and abundance in which outlier sampling events were included; (2) GLMMs for biomass excluding large-sized fish (defined as those > 3 standard deviations above the mean mass of all sampled fish); (3) GLMMs for abundance and richness excluding rare species (defined as those represented by < 4 individuals in all samples combined), dominant species (defined as the top 10% most numerically-abundant species in all samples combined), and both rare and dominant species (see Supplementary Table S5 online). In only one case (GLMM for abundance, with outliers included) were WW floodplain lakes no longer significantly greater than all other water/habitat type combinations.

## Discussion

Our results avoid confounding variation associated with geographical variation in species distributions to conclude that fish communities in whitewater systems are distinct from those in blackwater and clearwater systems. Assemblage structure is correlated most strongly to turbidity and conductivity, and to a lesser degree to pH. We propose that variation in species composition between the three biogeochemical water types arises, in part, because each water type contains sets of species adapted to characteristic ranges of physico-chemical conditions, habitat structure and floodplain vegetation type. Amazonian fishes are known to exhibit a tremendous diversity of physiological, morphological, and sensory adaptations to water chemistry—including for hypoxia tolerance^61,62^, ion regulation under conditions of varying pH and ion concentration^63,64^, vision, phototransduction, and visual signaling under varying optical conditions^12,65^, and, in the case of Gymnotiformes, electrolocation and electrocommunication in waters of varying conductivity^66^. Gradients of water type are therefore expected to act as filters to dispersal^32^, influence the outcome of competitive interactions^14^, and regulate genetic exchange between populations^67^—in turn leading to the accumulation of distinctive communities of fishes in different water types.

We noted that high-transparency black- and clearwaters, as well as whitewater lakes (where transparency increases with sediment precipitation), host a larger proportion of species belonging to orders with well-developed visual systems and diurnal activity, e.g. Characiformes, Cichliformes, and Perciformes. In contrast, low-transparency whitewater river waters host a larger proportion of species belonging to orders with reduced eye size, predominantly nocturnal activity, and sensory systems that function independently of light—notably the well-developed olfactory systems of Siluriformes, active electroreception in Gymnotiformes, and passive electroreception in Siluriformes, Gymnotiformes, Myliobatiformes, and Ceratodontiformes^66^. These observations suggest that adaptive trade-offs between visual and non-visual sensory systems may contribute to the distinct species assemblages in waters of different transparency. Physiological adaptations for ion regulation in low waters with low ion concentrations (low conductivity) and low pH have been well documented from fishes specialized to blackwaters^11,64,68,69^, and likely also play a strong role in structuring fish communities.

We also observed greater differences in community structure associated with water type than with habitat type (i.e. river versus floodplain) and postulate that the similarity of adjacent floodplain and river communities arises from lateral movements of fish across the river-floodplain ecotone. These movements are documented to occur in response to the seasonal availability of food^51,70,71^ and dissolved oxygen^62^, or for reproduction^72^.

In addition to influencing ion regulation physiology, variation in conductivity (a measure of dissolved nutrient content) between water types may exert indirect impacts on fish community composition by influencing aquatic vegetation. For instance, the dense floating macrophyte stands unique to whitewater floodplains host distinctive communities of fishes^70^, many of which we encountered only in our whitewater lake samples (especially small-bodied Characidae, Supplementary Table S3 online). Similar disparities in species composition associated with nutrient availability have been reported in Amazonian bird and mammal communities, where distinct assemblages are associated with differences in tree species composition and forest biomass on nutrient-rich versus nutrient-poor soils^73,74^.

One important limitation of our analyses of community composition is that we sampled only the marginal habitats of river channels, thus excluding deep benthic communities dominated by Gymnotiformes and Siluriformes^75,76^, as well as large open-water migratory Siluriformes and Characiformes^51^. Future work is needed to integrate these faunal components into comparisons of assemblage structure between Amazon water types.

### Species richness

Suggestive of a positive correlation between ecosystem productivity and species richness, we documented high mean (per sampling event) species richness in systems in habitats known to support high autochthonous and allochthonous PP (i.e. nutrient-rich sediment-decanted whitewater floodplain lakes), but lower mean species richness in habitats known to support lower PP (i.e. low-nutrient blackwater and clearwater rivers and floodplain lakes, and light-limited whitewater rivers). We acknowledge that we did not directly measure PP in our study sites. However, we did demonstrate that the physico-chemical conditions and other environmental parameters measured at our black-, clear-, and whitewater sites are typical for the basin and should conform to the well-documented disparities in ecosystem productivity summarized above (see “Introduction” for details).

Species-energy hypotheses predict elevated species richness under conditions of increased energy availability due to an increased number of trophic levels, heightened speciation rates (e.g. via metabolic effects on growth rates, generation times, and fecundity), or lowered extinction rates (e.g. via influences on effective populations sizes)^59^. Such relationships have been documented in freshwater habitats^77–79^ and are a well-described feature of terrestrial ecosystems^58,59^, including for Amazonian mammals^80^. However, the two previously published quantitative comparisons of fish species richness between Amazonian water types provided contradictory results; Saint-Paul et al.^31^ reported higher diversity in blackwaters than whitewaters, but Henderson and Crampton^30^ reported the opposite.

We hesitate to interpret our observations of mean species richness as evidence for a generalized species-energy relationship across Amazonian water types and habitats. In the first place, we reported the highest total, rarefied, and Chao1-estimated species richness in whitewater habitats of the mainstem R. Amazonas (with a basin size of ca. 6.3 × 10^6^ km^2^), followed by clearwater habitats of the smaller R. Tapajós sub-basin (ca. 2.4 × 10^5^ km^2^), and then by blackwater habitats of the much smaller R. Arapiuns sub-basin (ca. 1.8 × 10^3^ km^2^). Because freshwater fish species richness is known to correlate positively with river basin size^78^, including for the Amazon basin^7^, we cannot exclude the possibility that the water-type variation in species richness we report here is biased by basin-size effects. Second, our results may also be biased by the larger area of whitewater than black- and clearwater floodplains in our study area; whitewater floodplains are generally larger than the floodplains of black- or clearwater rivers of similar size because of their high sediment load^17,81^. Third, although we reported less than half the *mean* (per sampling event) species-richness in whitewater rivers than whitewater floodplains, total and rarified species richness, as well as other indices of species diversity were all higher in whitewater rivers than whitewater floodplains (we presume the discrepancy arises from a higher rate of seasonal species turnover in whitewater rivers than floodplains). Our mean per-sampling event estimates of species richness in whitewater rivers may therefore underestimate species richness, which in turn challenges the notion that the low autochthonous PP of this habitat is associated with low species richness. Fourth, the extent to which fish populations in rivers are subsidized by energy from adjacent floodplains is poorly known. For example, studies of terrestrial-aquatic trophic linkages in blackwater rivers have confirmed that allochthonous inputs from flooded forests are the dominant energy source for riverine fish^82^. Likewise, stable isotope analyses suggest that detritivorous characiform fish in whitewater rivers mostly assimilate carbon originating from phytoplankton (of floodplain origin), while siluriform whitewater riverine fish assimilate carbon from trees and C3 macrophytes (also of floodplain origin)^83^. Much of this carbon is thought to enter the rivers as detritus, which accumulates on the river bed and supports food chains based on heterotrophic bacteria^27^. If these allochthonous inputs are included, rivers (whitewater in particular) may have more energy available for fish populations than implied by the low levels of autochthonous PP. Finally, because we did not sample deep benthos-specialized riverine fish and large migratory species, our estimates of riverine species richness are incomplete.

In light of these limitations, establishing a generalized habitat-wide positive species-energy relationship for Amazonian fish will ultimately require quantitative comparisons of fish species richness between the three biogeochemical water types at a larger geographical scale. Future studies should ideally select sites in river basins of variable size as well as in floodplains of varying area, and would benefit from the inclusion of whitewater systems of the upper Amazon, which exhibit higher dissolved nutrient content than those of the lower Amazon^20,21,84^. Our results also highlight the need for a more complete understanding of the trophic pathways by which energy is transferred from autochthonous and allochthonous primary production to fishes.

A recent basin-wide model-based analysis of Amazon fish species richness^7^ reported a positive correlation between species richness and “energy availability”, when corrected for sub-basin size. However, in this study energy availability was defined by terrestrial net PP estimates from the WorldClim1 dataset, which are based on solar radiation and rainfall. We suspect that this approach may be only partially informative of species-energy relationships for Amazonian fish because the PP of Amazonian aquatic systems is determined by water biogeochemistry, which is not known to correlate to solar radiation or local rainfall across the basin.

### Biomass and abundance

Mirroring the patterns we report above for species richness, we documented greater mean per-sampling-event fish biomass in the systems with high ecosystem productivity (whitewater floodplains lakes) than in systems with relatively low PP (blackwater and clearwater lakes and rivers, and whitewater rivers). These relationships were not predicted a priori, because food chain models typically predict top-down trophic cascades in which elevated PP may increase consumer abundance and biomass at some trophic levels, but not others^85^. Moreover, evidence for positive correlations between PP and freshwater consumer biomass is fragmentary^86,87^. In Amazonian aquatic systems, Arbeláez et al.^88^ reported a strong positive correlation between water nutrient content and fish biomass in upland terra firme streams draining different geological formations. Saint-Paul et al.^31^ reported higher biomass in whitewaters than nearby blackwaters. However, Henderson and Crampton^30^ reported no year-round differences in biomass between blackwaters and adjacent whitewaters. Pereira et al.^89^ documented higher bat biomass (but lower species richness) in the inundation forests of nutrient-rich whitewater floodplains than in nutrient-poor blackwater floodplains.

Again, we are hesitant to interpret our result as evidence for a generalized positive energy-biomass relationship. First, our estimates of biomass were standardized by capture per unit effort (CPUE) but were not adjusted for habitat area. Second, we recall that our sampling program excluded deep river and large migratory river species, and therefore omitted an important component of riverine biomass. Nonetheless, our observations of higher biomass in whitewater floodplains than black- and clearwater habitats are consistent with the fact that whitewater floodplains have supported large commercial fisheries in the central and lower Brazilian Amazon since the 1970s, while no major commercial fishing operations occur in blackwater and clearwater river-floodplain systems—even where these form large areas near major cities (e.g. the middle-lower Rio Negro)^29,90,92^. Disentangling the effects of ecosystem productivity and habitat area on fish biomass and fisheries production will require sampling floodplains of variable size from all three water types, over a wider geographical area.

In conclusion, our study represents the first direct quantitative comparison of the fish faunas of the three Amazonian water types first recognized over 160 years ago by Wallace. We demonstrate for the first time that whitewater fish assemblages are distinct from the fish assemblages of oligotrophic blackwater and clearwater systems. Our study also provides novel perspectives on how PP, determined mainly by dissolved nutrient content, sediment type, water transparency, and acidity, may influence fish assemblage structure within a single megadiverse biome. Our results provide some support for a positive relationship between ecosystem productivity and both species richness and biomass. However, to confirm whether these energy richness/biomass relationships are generalized across all Amazonian water types and river-floodplain habitats, while excluding sub-basin and habitat size effects, a sampling program covering a much larger geographical area will ultimately be required. The freshwater habitats and fish biodiversity of the Amazon face unprecedented pressure from habitat degradation, over-fishing, and climate change^91^. Our study highlights the importance of including multiple water types and habitats in conservation monitoring and planning. Nonetheless, long-term strategies for aquatic biodiversity protection will require a fuller understanding of the effects of habitat and water type on fish diversity and distributions.

## Materials and methods

### Sampling

For the BW Arapiuns, CW Tapajós, and WW Amazonas rivers (Fig. 2), we sampled repeatedly at two sites (‘site replicates’ R1, R2) on the main river channel margin and two adjacent floodplain lake sites (site replicates F1, F2). Our sampling design therefore comprised six ‘water type’/’habitat type’ combinations, each replicated twice to yield 12 repeatedly sampled sites. Our sampling sites (Fig. 2) were located ca. 25 km upstream of a zone of seasonal mixing at the three-way Arapiuns-Tapajós and Tapajós-Amazonas confluence, but within the range over which most fish species would be expected to freely disperse in the absence of barriers. We selected perennial floodplain lakes (those with permanent water content for 30 + years, based on historical satellite images) of similar area (blackwater F1 = 0.17 km^2^, F2 = 0.25 km^2^; clearwater F1 = 0.18 km^2^, F2 = 0.21 km^2^; whitewater F1 = 0.18 km^2^, F2 = 0.26 km^2^). Sampling was conducted through a full year (beginning October 2014), including during low-water (October, December, February) and high-water (April, June, August), for a planned total of 72 sample events (one WW river sampling event was missed due to storms, leaving 71). For each sampling event we measured temperature, pH, dissolved oxygen, and conductivity with a Hanna HI-9829 meter, and turbidity with a LaMotte 2020 meter, always at 6 am, 10 cm depth, and 10 m from the shore. To maximize the diversity of sampled fish we utilized two ‘gear types’, gill and seine nets, each of which is selective of different species and body sizes. Gill nets were deployed in batteries of four (25 × 3 m, 15, 30, 45, 60 mm mesh) from 6 to 9 am and from 6 to 9 pm for every sampling event, thereby targeting species with both diurnal and nocturnal activity. Seine netting was conducted during the low-water months only—when the net could reach the bottom. We deployed a 30 × 3 m net with 5 mm mesh three times between 6 and 9 am, and three times between 6 and 9 pm. In the low-water months we pooled the results of the gill and seine nets. We emphasize that CPUE sampling effort over the year was identical for the three water types and two habitats—permitting equitable comparisons between all water type and habitat type combinations (but not between seasons). Fish were euthanized with 600 mg L^−1^ eugenol, fixed in 10% formalin, and preserved in 70% EtOH. Our collections were deposited at the biodiversity collections listed in Supplementary Appendix S3 online and identified using descriptions and keys from the taxonomic literature on Amazonian fish; see van der Sleen and Albert^92^ for recent review. Field sampling was authorized by the Brazilian Conselho Nacional de Desenvolvimento Científico e Tecnológico (Scientific Expedition 02448/2012-2) and Instituto Chico Mendes de Conservação da Biodiversidade (permits 37742-1-4). Animal use was approved by the Comissão de Ética no Uso de Animais, Pontifícia Universidade Católica, Rio Grande do Sul (Protocol 15/00,456), and by the University of Central Florida Institutional Animal Care and Use Committee (Protocols 12–31 W, 15–24 W).

### Data conditioning and statistical analysis

All procedures were performed in R^93^ using functions from vegan^94^ and MASS^95^, unless otherwise stated.

#### Physico-chemical parameters

We compiled a matrix of physico-chemical properties, henceforth ‘abiotic matrix’, at each of the 71 sampling events, conditioned by unity-based normalization. We used PERMANOVA to test for differences in physico-chemical properties between water types, and PCA to visualize groupings of sampling events by physico-chemical properties.

#### Comparisons of species richness, biomass, and abundance between water types and habitats

We compiled a matrix of the abundances of all species at each of the 71 sampling events, henceforth ‘biotic matrix’, from which we calculated the descriptors of richness and diversity presented in Table 1. We then performed the following three sets of analyses: (1) GLMMs, using the R package lme4^96^ to evaluate disparities in species richness, biomass, and abundance as a function of habitat nested inside water type, and with ‘time of the year’ (month) and ‘site replicate’ (the two replicates of each water type/habitat combinations) assigned as random effect variables, e.g. biomass ~ water type/habitat type + (1|time of year) + (1|site replicate). We specified negative binomial distributions (log link) for abundance and species richness, and Gamma distributions (log link) for biomass. We confirmed that residual distributions satisfied model assumptions. Finally, we calculated the least square means and associated 95% confidence intervals (CIs) for each habitat/water type combination using the R package emmeans^97^, and tested the significance of differences by Tukey’s HSD. (2) NMDS based on ranked Bray–Curtis dissimilarity indices of square root-transformed species abundances, with five dimensions, to visualize differences in assemblage structure between water types and habitats. (3) PERMANOVA to test for differences in assemblage structure between water types. We also used the PERMANOVA coefficients as indices of the relative contributions of the 20 most influential species to the differences among the three pairs of water types.

#### Correlation of assemblage structure to physico-chemical properties

We performed co-inertia analysis (COIA) to assess the correlation between a Principal Coordinate Analysis (PCoA) of Bray–Curtis distances in the biotic matrix and a PCA of the abiotic matrix. A Monte Carlo-based permutation test estimated significance of the coefficient of correlation (RV value) from this COIA. We also generated a null model based on 1,000 randomizations of the biotic matrix (with species shuffled between water/habitat types but row and column totals constrained) from which we derived a mean COIA RV value with 95% CIs. We assumed observed RV values outside the null 95% CIs did not arise by chance.

## Supplementary information

Supplementary Appendix 1.

Supplementary Appendices 2–5.

Supplementary Tables.

Supplementary Figures.

## Data Availability

All data in support of the results presented herein are provided in supplementary documents.
