# Supplementary material for: Biogeochemical water type influences community composition, species richness, and biomass in megadiverse Amazonian fish assemblages
Source: Sci Rep. 2020 Sep 18;10:15349. doi: 10.1038/s41598-020-72349-0 (PMC7501869; doi:10.1038/s41598-020-72349-0)
Supplement: Supplementary file 2 — Supplementary Appendices 2–5. [file 41598_2020_72349_MOESM2_ESM.pdf]

Online Resource

Supplementary Appendices 2-5

Biogeochemical water type influences community composition, species richness, and biomass in megadiverse Amazonian fish assemblages

Juan David Bogotá-Gregory, Flávio C. T. Lima, Sandra B. Correa, Cárlison Silva-Oliveira, David G. Jenkins, Frank R. Ribeiro, Nathan R. Lovejoy, Roberto E. Reis, William G. R. Crampton*

* Corresponding author: Department of Biology, University of Central Florida, 4100 Libra Dr., 32816 Orlando, FL, USA. Email: crampton@ucf.edu

Appendix S2

Spatial dependence analysis

Methods: In the main text (Materials and methods: Correlation of assemblage structure to physico-chemical properties) we performed a co-inertia analysis (COIA, henceforth COIA-1) to determine the extent to which species abundances and physico-chemical water parameters are correlated. To determine if the relationship between species abundances and physico-chemical water parameters has spatial dependence, we calculated the hydrological distance between all pairs of sites (using the Path tool in Google Earth) and incorporated these into a principal component of neighbor distances (PCNM) among all 71 sample events (henceforth 'distance matrix'). We then merged this distance matrix with the principal component analysis (PCA) of the 'abiotic matrix' described in the main text. Finally, we correlated this merged matrix to the (PCoA) of the Bray-Curtis indices of the biotic matrix described in the main text, via a new COIA (henceforth COIA-2). The magnitude of the difference between the RV of COIA-1 and COIA-2 (Δ RV) informed us of the degree of spatial dependence, with a low Δ RV indicating low spatial dependence.

Results:

COIA-2 yielded an RV of 0.47 ($P < 0.001$; null model 95% CIs 0.108–0.115). The low Δ RV (0.01) between COIA-1 (RV = 0.46 – see Main text – Results – Community composition) and COIA-2 indicates that the relationship between assemblage structure and physico-chemical parameters has negligible spatial dependence, i.e. was uninfluenced by the distances between the three sets of sites.

Appendix S3

Biodiversity collections hosting collected fishes

Excepting some large specimens belonging to common, readily-identifiable species, our entire collection was cataloged at Museu de Ciências e Tecnologia, Pontifícia Universidade Católica do Rio Grande do Sul (MCP), Universidade Federal do Oeste do Pará (UFOPA), and Museu de Zoologia da Universidade Estadual de Campinas (ZUEC) in Brazil, and Florida Museum of Natural History (UF) in the USA.

Appendix S4

Literature supporting ranges of physico-chemical water properties for Fig. 1

Affonso AG, Barbosa C, Novo EMLM (2011a) Water quality changes in floodplain lakes due to the Amazon river flood pulse: Lago Grande de Curuaí (Pará). Braz. J. Biol. 71:601-610

Affonso AG, de Queiroz HL, Novo EMLM (2011b) Limnological characterization of floodplain lakes in Mamirauá Sustainable Development Reserve, central Amazon (Amazonas State, Brazil). Acta Limnol. Brasil. 23:95-108

Arantes CC, Castello L, Cetra M, Schilling A (2013) Environmental influences on the distribution of arapaima in Amazon floodplains. Environ. Biol. Fishes 96:1257–1267

Barthem R, Goulding M, Fosberg BR, Cañas C, Ortega H (2003) Aquatic ecology of the Rio Madre de Dios: Scientific bases for Andes-Amazon headwater conservation. Amazon Conservation Association, Lima, Peru

Borges Pedro JP, Gomes MCRL, Jesus Trindade ME, Cavalcante DP, Oliveira JA, Hercos AP, Zucchi N, Lima CB, Pereira SA, Queiroz HL (2013) Influence of the hydrological cycle on physical and chemical variables of water bodies in the várzea areas of the middle Solimões river region (Amazonas, Brazil). Uakari 9:33-47

Bozelli RL (1992) Composition of the zooplankton community of Batata and Mussurá lakes of the Trombetas River, state of Pará, Brazil. Amazoniana 12:239-261

Brinkmann WL, Santos UM (1973) Heavy fish kill in unpolluted floodplain lakes of Central Amazonia, Brazil. Biol. Conserv. 5:147-149

Brito JG, Alves LF, Espirito Santos HMV (2014) Seasonal and spatial variations in limnological conditions of a floodplain lake (Lake Catalão) connnected to both the Solimões and Negro rivers, central Amazonia. Acta Amazon. 44:121-134

Caraballo P, Forsberg BR, Almeida FF, Leite RG (2014) Diel patterns of temperature, conductivity and dissolved oxygen in an Amazon floodplain lake: description of a *friagem* phenomenon. Acta Limnol. Brasil. 26:318-331

Carvalho P, Bini LM, Thomaz SM, Goncalves de Oliveira L, Robertson B, Tavechio WLG, Darwisch AJ (2001) Comparative limnology of South American floodplain lakes and lagoons. Acta Sci. 23:265-273

Coelho-Souza SA, Guimarães PR, Miranda MR, Poirier H, Mauro JBN, Lucotte M, Mergler D (2011) Mercury and flooding cycles in the Tapajós river basin, Brazilian Amazon: The role of periphyton of a floating macrophyte (Paspalum repens). Sci. Total Environ. 409:2746-2753

Correa SB (2008) Fish assemblage structure is consistent through an annual hydrological cycle in habitats of a floodplain-lake in the Colombian Amazon. Neotropical Ichthyology 6:257-266

Crampton WGR (1998) Electric signal design and habitat preferences in a species rich assemblage of gymnotiform fishes from the upper Amazon basin. Anais da Academia Brasileira de Ciências 70:805-847

Crampton WGR (2008) Ecology and life history of an Amazon floodplain cichlid: the discus fish *Symphysodon* (Perciformes: Cichlidae). Neotropical Ichthyology 6:599-612

Crampton WGR (2011) An ecological perspective on diversity and distributions In: Albert JS, Reis RE (eds) Historical biogeography of neotropical freshwater fishes. University of California Press, Berkeley, pp 165-189

Crampton WGR, Chapman LJ, Bell J (2008) Interspecific variation in gill size is correlated to ambient dissolved oxygen in the Amazonian electric fish *Brachyhypopomus* (Gymnotiformes: Hypopomidae). Environ. Biol. Fishes 83:223-235. doi: 10.1007/s10641-007-9325-3

Crampton WGR, De Santana CD, Waddell JC, Lovejoy NR (2016a) Phylogenetic systematics, biogeography, and ecology of the electric knifefish genus *Brachyhypopomus* (Ostariophysi: Gymnotiformes). PLOS One 11 (e0161680):1-66

Crampton WGR, de Santana CD, Waddell JC, Lovejoy NR (2016b) A taxonomic revision of the Neotropical electric fish genus *Brachyhypopomus* (Ostariophysi: Gymnotiformes: Hypopomidae), with descriptions of 15 new species. Neotropical Ichthyology 14:639-790

Crampton WGR, Lovejoy NR, Albert JS (2003) *Gymnotus ucamara*: a new species of Neotropical electric fish from the Peruvian Amazon (Ostariophysi: Gymnotidae), with notes on ecology and electric organ discharges. Zootaxa 277:1-18

Darwich AJ, Aprile FM, Robertson BA (2005) Variáveis limnológicas: contribuição ao estudo espaço-temporal de águas pretas amazônicas. In: Santos-Silva EN, Aprile FM, Scudeller VV, Melo S (eds) BioTupé: meio físico, diversidade biológica e sociocultural do baixo rio Negro Amazônia central. Instituto Nacional de Pesquisas da Amazônia, Manaus, pp 20-33

de Oliveira EC, Ferreira EJG (2008) Spawning areas, dispersion and microhabitats of fish larvae in the Anavilhanas Ecological Station, rio Negro, Amazonas State, Brazil. Neotropical Ichthyology 6:559-566

de Queiroz HL (2007) Classification of water bodies based on biotic and abiotic parameters at the várzeas of Mamirauá Reserve, Central Amazon. Scientific Magazine UAKARI 3:19-34

de Queiroz LJ, Torrente-Vilara G, Vieira FG, Ohara WM, Zuanon J, Doria CRC (2013) Fishes of Cuniã Lake, Madeira River Basin, Brazil. Check List: Journal of Species Lists and Distribution 9:540-548

Devol AH, Hedges JI (2001) Organic matter and nutrients in the mainstem Amazon River. In: McClain ME, Victoria RL, Richey JE (eds) The biogeochemistry of the Amazon basin. Oxford University Press, Oxford, pp 275-306

Duncan WP, Fernandes MN (2010) Physicochemical characterization of the white, black, and clearwater rivers of the Amazon Basin and its implications on the distribution of freshwater stingrays (Chondrichthyes, Potamotrygonidae). Pan-American Journal of Aquatic Sciences 5:454-464

Dustan P (2009) Terrestrial limitation of Amazon river productivity: why the Amazon River is not green. Evol. Ecol. Res. 11:421-432

Furch K (1984) Water chemistry of the Amazon basin: the distribution of chemical elements among fresh waters. In: Sioli H (ed) The Amazon: Limnology and landscape ecology of a mighty tropical river and its basin. Dr W. Junk, Dordrecht, pp 168-199

Furch K, Junk WJ, Dieterichf J, Kochert N (183) Seasonal variation in the major cation (Na, K, Mg an Ca) content of the waterof the Lago Camaleão, an Amaonian floodplain-lake near Manaus, Brazil. Amazoniana 8:75-89

Galacatos K, Barriga-Salazar R, Stewart DJ (2004) Seasonal and habitat influences on fish communities within the lower Yasuni River basin of the Ecuadorian Amazon. Environ. Biol. Fishes 71:33-51. doi: 10.1023/B:EBFI.0000043156.69324.94

Geisler R, Schneider J (1976) The element matrix of Amazon waters and its relationship with the mineral content of fishes (determination using Neutron Activation Analysis). Amazoniana 6:47-65

Gibbs RJ (1972) Water chemistry of the Amazon river. Geochim. Cosmochim. Acta 36:1061-1066. doi: 10.1016/0016-7037(72)90021-X

Hardy ER (1980) Composição do zooplâncton em cinco lagos da Amazônia Central. Acta Amazon. 10:577-609

Henderson PA, Crampton WGR (1997) A comparison of fish diversity and abundance between nutrient-rich and nutrient-poor lakes in the Upper Amazon. J. Trop. Ecol. 13:175-198. doi: 10.1017/S0266467400010403

Horbe A, Santos AG (2009) Chemical composition of black-watered rivers in the western Amazon Region (Brazil). Journal of the Brazilian Chemical Society 20:1119-1126

Junk WJ (1970) Investigations on the ecology and production-biology of the "floating meadows" (*Paspalo-Echinochloetum*) on the middle Amazon. Part I: The floating vegetation and its ecology. Amazoniana 2:449-495

Junk WJ (1973) Investigations on the ecology and production-biology of the "floating meadows" (*Paspalo-Echinochloetum*) on the middle Amazon. Part II. The aquatic fauna in the root zone of floating vegetation. Amazoniana 4:9-102

Junk WJ (1980) Química da água e macrófitas aquáticas de rios e igarapés na bacia Amazônica e nas áreas adjacentes. Acta Amazon. 10:611-633

Junk WJ (1997) General aspects of floodplain ecology with special reference to Amazonian floodplains. In: Junk WJ (ed) The Central Amazon floodplain: ecology of a pulsing system. Springer-Verlag, Berlin, pp 3-20

Junk WJ, Furch K (1985) The physical and chemical properties of the Amazonian waters and their relationships with the biota. In: Prance GT, Lovejoy TE (eds) Amazonia. Pergamon/IUCN, Oxford, pp 3-17

Junk WJ, Piedade MTF (2010) An introduction to South American wetland forests: distribution, definitions and general characterization. In: Junk WJ, Piedade MTF, Wittmann F, Schöngart J, Parolin P (eds) Amazonian floodplain forests: Ecophysiology, biodiversity and sutainable management. Springer, Dordrecht, pp 3-25

Junk WJ, Piedade MTF, Schöngart J, Cohn-Haft M, Adeney JM, Wittman F (2011) A classification of major naturally-occurring Amazonian lowland wetlands. Wetlands 31:623-640. doi: 10.1007/s13157-011-0190-7

Junk WJ, Teresa T, Piedade F, Schöngart J, Wittmann F (2012) A classification of major natural habitats of Amazonian white-water river floodplains (várzeas). Wetlands Ecol. Manage. 20:461-475. doi: 10.1007/s11273-012-9268-0

Leite NK, Krusche AV, Ballester MV, Victoria RL, Richey JE, Gomes BM (2011) Intra and interannual variability in the Madeira River water chemistry and sediment load. Biogeochemistry 105:37-51

Marlier G (1967) Ecological studies on some lakes of the Amazon valley. Amazoniana 1:91-115

Melack JM (1984) Amazon floodplain lakes: shape, fetch, and stratification. Verhandlungen des Internationalen Verein Limnologie 22:1278-1282

Melack JM, Fisher TR (1983) diel oxygen variations and their ecological implications in Amazon floodplains. Archiv Für Hydrobiologie 98:422-442

Melack JM, Forsberg BR (2001) Biogeochemistry of Amazon floodplain lakes and associated wetlands. In: McClain ME, Victoria RL, Richey JE (eds) The biogeochemistry of the Amazon basin. Oxford University Press, Oxford, pp 235-274

Petry AC, Agostinho AA, Gomes LC (2003a) Fish assemblages of tropical floodplain lagoons: exploring the role of connectivity in a dry year. Neotropical Ichthyology 1:111-119

Petry P, Bayley PB, Markle DF (2003b) Relationships between fish assemblages, macrophytes and environmental gradients in the Amazon River floodplain. J. Fish Biol. 63:547-579. doi: 10.1046/j.1095-8649.2003.00169.x

Rai H, Hill G (1980) Classification of Central Amazon lakes on the basis of their microbiological and physico-chemical characteristics. Hydrobiologia 72:85-99

Rai H, Hill G (1981) Physical and chemical studies of Lago Tupi: a Central Amazon black water ria lake. Int. Revue. Ges. Hydrobiol. 66:37-82

Reiss F (1977) Qualitative and quantitative investigations on the macrobenthic fauna of central Amazon lakes. 1. Lago Tupé, a blackwater lake on the lower rio Negro. Amazoniana 6:203-235

Richey JE, Victoria RL, Salati E, Fosberg BR (1991) The biogeochemistry of a major river system: the Amazon case study. In: Degens ET, Kempe S, Richey JE (eds) Biogeochemistry of Major World Rivers. John Wiley and Sons Ltd., New York, pp 57-74

Ríos-Villamizar E, Piedade M, Da Costa J, Adeney J, Junk W (2013) Chemistry of different Amazonian water types for river classification: a preliminary review. Water and society ii 178:1117

Röpke CP, Amadio SA, Winemiller KO, Zuanon J (2016) Seasonal dynamics of the fish assemblage in a floodplain lake at the confluence of the Negro and Amazonas rivers. J. Fish Biol. 89:194-212. doi: 10.1111/jfb.12791

Sánchez-Botero JI, Araujo-Lima CARM, Garcez DS (2008) Effects of types of aquatic macrophyte stands and variations of dissolved oxygen and of temperature on the distribution of fishes in lakes of the amazonian floodplain. Acta Limnol. Brasil. 20:45-54

Sánchez-Botero JI, de Farias ML, Piedade MT, Garcez DS (2003) Ictiofauna associada às macrófitas aquáticas *Eichhornia azurea* (SW.) Kunth. e *Eichhornia crassipes* (Mart.) Solms. no lago Camaleão, Amazônia central, Brasil. Acta Scientiarum Biological Sciences 25:369-375

Sánchez-Botero JI, Garcez DS, Lobón-Cerviá J (2001) Oxigênio dissolvido e temperatura em lagos da região de Ati Paraná-Solimões, Amazônia Central, Brasil. Acta Limnol. Brasil. 13:45-51

Saul WG (1975) An ecological study of fishes at a site in upper Amazonian Ecuador. Proceedings Academy of Natural Sciences of Philadelpia 127:93-134

Schmidt GW (1973a) Primary production of phytoplankton in the three types of Amazonian waters II. The limnology of a tropical floodplain lake in Central Amazonia (Lago do Castanho). Amazoniana 4:139-203

Schmidt GW (1973b) Primary production of phytoplankton in the three types of Amazonian waters III. Primary productivity of phytoplankton in a tropical floodplain lake of Central Amazonia, Lago do Castanho, Amazonas, Brazil. Amazoniana 4:379-404

Schmidt GW (1973c) Studies on the primary productivity of phytoplankton in the three types of Amazonian waters. I. Introduction. Amazoniana 4:135-138

Schmidt GW (1976) Primary production of phytoplankton in the three types of Amazonian waters. IV. On the primary productivity of phytoplankton in a bay of the lower Rio Negro (Amazonas, Brazil). Amazoniana 5:517-528

Schmidt GW (1982) Primary production of phytoplankton in the three types of Amazonian waters V. Some investigations on the phytoplankton and its primary productivity in the clear water of the lower Rio Tapajóz (Pará, Brazil). Amazoniana 7:335-348

Seyler PT, Boaventura GR (2001) Trace elements in the mainstem Amazon river. In: McClain ME, Victoria RL, Richey JE (eds) The biogeochemistry of the Amazon basin. Oxford University Press, Oxford

Sioli H (1950) Das Wasser in Amazonasgebiet. Forsch. Fortschr. 26:274-280

Sioli H (1967) Studies in Amazonian waters. Atlas do simpósio sobre a biota amazônica (Limnologia) (Vol. 3). Conselho Nacional de Pesquisas, Rio de Janeiro, pp 9-50

Sioli H (1984) The Amazon and its main affluents: Hydrography, morphology of the river courses, and river types. In: Sioli H (ed) The Amazon: Limnology and landscape ecology of a mighty tropical river and its basin, vol 56. Junk, Dordrecht, pp 127-165

Tejerina-Garro FL, Fortin R, Rodriguez MA (1998) Fish community structure in relation to environmental variation in floodplain lakes of the Araguaia River, Amazon Basin. Environ. Biol. Fishes 51:399-410

Thomé-Souza MJF, Chao NL (2004) Spatial and temporal variation of benthic fish assemblages during the extreme drought of 1997-9 (El Nino) in the middle rio Negro, Amazonia, Brazil. Neotropical Ichthyology 2:127-136

Trevisan GV, Jardim-Lima DJ (2012) Relationship between water transparency and physical-chemical variables in lakes of the western Amazon, Brazil. Acta Scientiarum. Biological Sciences 34:13-21

Tundisi JG, Forsberg BR, Devol AH, Zaret TM, Tundisi TM, Santos A, Ribeiro JS, Hardy ER (1984) Mixing patterns in Amazon lakes. Hydrobiologia 108:3-15

Appendix S5

Designation of fish orders to predominantly diurnal versus nocturnal activity:

Osteoglossiformes: Diurnal (Agudelo-Zaomora et al. 2007).

Clupeiformes: Diurnal (Rodriguez and Lewis 1997; Arrington and Winemiller 2003; Arantes et al. 2018)

Characiformes: Diurnal (Roberts 1972) (Lowe-McConnell 1975; Rodriguez and Lewis 1997; Tejerina-Garro et al. 1998; Galacatos et al. 2004; Carvalho et al. 2007; Brejão et al. 2013).

Gymnotiformes: Nocturnal (Crampton 2019).

Siluriformes: Nocturnal (Lowe-McConnell 1975; Rodriguez and Lewis 1997; Tejerina-Garro et al. 1998; Carvalho et al. 2007; Brejão et al. 2013; Arantes et al. 2018).

Gobiiformes (*Microphylipnus ternetzi*): Diurnal (Flavio T. Lima. pers. obs).

Synbranchiformes: Diurnal (Junges et al. 2007)

Pleuronectiformes: Diurnal (Arrington and Winemiller 2003).

Cyprinodontiformes: (Arrington and Winemiller 2003; Brejão et al. 2013).

Beloniformes: Diurnal (Goulding and Carvalho 1983).

Tetraodontiformes: Diurnal (Krumme et al. 2007) (and pers. obs. for *Colomesus asellus*)

Cichliformes: Diurnal (Lowe-McConnell 1969; Lowe-McConnell 1975; Rodriguez and Lewis 1997; Tejerina-Garro et al. 1998; Carvalho et al. 2007; Arantes et al. 2018)

Perciformes: Diurnal (Arrington and Winemiller 2003)

Myliobatiformes: Nocturnal. Pers. obs; (Lasso et al. 1996).

Ceratodontiformes: Diurnal (Gonzalez Naya et al. 2008).

References

Agudelo-Zamora H, López-Macias JN, Sánchez Páez CL (2007) Hábitos alimentarios de la arawana (*Osteoglossum bicirrhosum* Vandelli, 1829) (Pisces: Osteoglossidae) en el alto río Putumayo, área del Parque Nacional Natural La Paya, Putumayo, Colombia. Rev. Elec. Ing. Prod. Acuic. 2:93-98

Arantes CC, Winemiller KO, Petrere M, Castello L, Hess LL, Freitas CEC (2018) Relationships between forest cover and fish diversity in the Amazon River floodplain. J. Appl. Ecol. 55:386-395

Arrington DA, Winemiller KO (2003) Diel changeover in sandbank fish assemblages in a neotropical floodplain river. J. Fish Biol. 63:442-459.

Brejão GL, Gerhard P, Zuanon J (2013) Functional trophic composition of the ichthyofauna of forest streams in eastern Brazilian Amazon. Neotrop. Ichthyol. 11:361-373.

Carvalho LN, Zuanon J, Sazima I (2007) Natural history of amazon fishes. In: Del Claro K, Oliveira PS, Rico-Gray V (eds) Tropical biology and conservation management: Case Studies (Volume XI). Eolss, Oxford, UK, pp 113-144

Crampton WGR (2019) Electroreception, electrogenesis and signal evolution. J. Fish Biol. 95:92-134.

Galacatos K, Barriga-Salazar R, Stewart DJ (2004) Seasonal and habitat influences on fish communities within the lower Yasuni River basin of the Ecuadorian Amazon. Environ. Biol. Fishes 71:33-51

Gonzalez Naya MJ, Volpedo AV, Gómez SE, Carignano L, Sánchez MV (2008) Notas sobre craneo, otolitos y biología de *Lepidosiren paradoxa* (Pisces, Dipnoi). Biol. Acuatica 24: no pagination.

Goulding M, Carvalho ML (1983) Ecology of Amazonian needlefishes (Belonidae). Rev. Bras. Zool. 2:99-111.

Junges CM, Lajmanovich RC, Peltzer PL, Attedemo AM, Bassó A (2007) Predator–prey interactions between *Synbranchus marmoratus* (Teleostei: Synbranchidae) and *Hypsiboas pulchellus* tadpoles (Amphibia: Hylidae): Importance of lateral line in nocturnal predation and effects of fenitrothion exposure. Chemosphere 81:1233-1238

Krumme U, Keuthen H, Saint-Paul U, Villwock W (2007) Contribution to the feeding ecology of the banded puffer fish *Colomesus psittacus* (Tetraodontidae) in north Brazilian mangrove creeks. Braz. J. Biol. 67:383-392.

Lasso CA, Rial A, Lasso-Alcalá O (1996) Notes on the biology of the freshwater stingrays *Paratrygon aiereba* (Müller y Henle, 1841) and *Potamotrygon orbignyi* (Castelnau, 1855) (Chondrichthyes: Potamotrygonidae) in the Venezuelan Llanos. J. Ichthyol. Aq. Biol. 2:39-50

Lowe-McConnell RH (1969) The cichlid fishes of Guyana, South America, with notes on their ecology and breeding behaviour. Zool. J. Linn. Soc. 48:255-302

Lowe-McConnell RH (1975) Fish communities in tropical freshwaters. Longman, London & New York

Roberts TR (1972) Ecology of fishes in the Amazon and Congo basins. Bull. Mus. Comp. Zool. 143:117-147

Rodriguez MA, Lewis WM (1997) Structure of fish assemblages along environmental gradients in floodplain lakes of the Orinoco River. Ecol. Monogr. 67:109-128

Tejerina-Garro FL, Fortin R, Rodriguez MA (1998) Fish community structure in relation to environmental variation in floodplain lakes of the Araguaia River, Amazon Basin. Environ. Biol. Fishes 51:399-410
