# Supplementary material for: Biogeochemical water type influences community composition, species richness, and biomass in megadiverse Amazonian fish assemblages
Source: Sci Rep. 2020 Sep 18;10:15349. doi: 10.1038/s41598-020-72349-0 (PMC7501869; doi:10.1038/s41598-020-72349-0)
Supplement: Supplementary file 3 — Supplementary Tables. [file 41598_2020_72349_MOESM3_ESM.pdf]

Online Resource

Supplementary Tables

Biogeochemical water type influences community composition, species richness, and biomass in megadiverse Amazonian fish assemblages

Juan David Bogotá-Gregory, Flávio C. T. Lima, Sandra B. Correa, Cárlison Silva-Oliveira, David G. Jenkins, Frank R. Ribeiro, Nathan R. Lovejoy, Roberto E. Reis, William G. R. Crampton*

* Corresponding author: Department of Biology, University of Central Florida, 4100 Libra Dr., 32816 Orlando, FL, USA. Email: crampton@ucf.edu

Table S1. Physico-chemical parameters for the blackwater, clearwater, and whitewater waters of the Santarém region, divided by habitat type and season.

Water type	Habitat	Season	Temperature (°C)			pH			DO (mg·L^{-1})		
			Min.	Max.	Mean	Min.	Max.	Mean	Min.	Max.	Mean
Blackwater	Floodplain lake	Low	29.3	31.2	30.2	4.3	5.6	4.8	2.7	8.3	5.7
		High	29.6	30.8	30.3	4.8	6.4	5.5	3.9	4.7	4.3
	River margin	Low	29.0	32.5	30.5	4.8	6.2	5.7	4.7	11.5	8.1
		High	29.2	30.6	30.1	5.0	6.0	5.5	4.5	9.1	5.5
Clearwater	Floodplain lake	Low	29.7	31.7	30.5	4.3	7.2	5.9	4.2	12.0	8.9
		High	29.3	30.6	30.0	5.4	7.0	6.1	3.0	4.5	4.0
	River margin	Low	29.1	30.7	30.1	6.3	7.3	6.8	5.2	11.3	8.6
		High	29.3	31.0	29.9	5.6	7.4	6.2	4.2	5.4	4.7
Whitewater	Floodplain lake	Low	28.6	32.0	30.0	6.9	7.8	7.2	0.7	9.2	5.0
		High	28.7	34.4	30.2	5.5	7.7	6.7	0.4	2.7	1.5
	River margin	Low	29.3	31.2	30.1	7.0	7.4	7.1	3.4	9.3	6.5
		High	28.0	30.4	29.1	5.7	7.0	6.3	2.3	2.8	2.6

Water type	Habitat	Season	Conductivity (μS·cm^{-1})			Turbidity (NTU)		
			Min.	Max.	Mean	Min.	Max.	Mean
Blackwater	Floodplain lake	Low	7.0	9.6	8.1	0.3	2.1	1.2
		High	7.5	9.8	8.5	1.3	2.0	1.6
	River margin	Low	7.5	11.2	9.1	0.1	7.6	2.7
		High	7.8	9.9	8.8	1.3	2.1	1.7
Clearwater	Floodplain lake	Low	4.4	13.4	10.0	3.6	13.5	6.6
		High	13.3	15.7	14.4	2.5	7.1	4.9
	River margin	Low	11.8	16.1	14.8	0.2	8.0	4.7
		High	13.8	16.1	14.8	2.0	9.6	6.0
Whitewater	Floodplain lake	Low	78.1	123.4	102.9	10.7	169.0	88.1
		High	53.5	81.5	60.3	13.1	45.6	31.2
	River margin	Low	46.7	64.5	54.3	27.9	305.0	129.3
		High	52.7	61.0	56.5	44.9	89.0	64.4

Table S2. Permutational multivariate analysis of variance (PERMANOVA) comparisons of physico-chemical water parameters between blackwater (BW), clearwater (CW), and whitewater (WW) systems of the Santarém region. All comparisons are significant at $\alpha = 0.001$. DF = degrees of freedom.

Comparison	River Margins (RM)			Floodplain lakes (FL)			Pooled RM and FL		
	Pseudo *F*	R	DF	Pseudo *F*	R	DF	Pseudo *F*	R	DF
Global (BW-CW=WW)	20.9	0.75	2, 33	24.1	0.77	2, 34	38.4	0.73	2, 68
BW-CW	5.4	0.45	1, 22	3.5	0.37	1, 22	7.2	0.36	1, 46
BW-WW	33.1	0.78	1, 21	43.9	0.81	1, 22	67.9	0.77	1, 45
CW-WW	22.3	0.71	1, 21	25.0	0.73	1, 22	42.0	0.69	1, 45

Table S3. List of sampled fish species[1]. BW = blackwater. CW = clearwater. WW = whitewater. RM = river margin. FL = floodplain lake. cf. = conferre.

Taxon	River Margin (RM)			Floodplain lake (FL)			RM & FL			RM	FL
	BW	CW	WW	BW	CW	WW	BW	CW	WW		
**Order Myliobatiformes**											
**Family Potamotrygonidae**											
Potamotrygon motoro (Müller & Henle 1841)	0	0	0	0	1	2	0	1	2	0	3
Potamotrygon orbignyi (Castelnau 1855)	0	1	0	0	8	0	0	9	0	1	8
Potamotrygon scobina Garman 1913	0	1	0	0	0	0	0	1	0	1	0
**Order Osteoglossiformes**											
**Family Osteoglossidae**											
Osteoglossum bicirrhosum (Cuvier 1829)	0	0	1	0	0	2	0	0	3	1	2
**Order Clupeiformes**											
**Family Clupeidae**											
Rhinosardinia amazonica (Steindachner 1879)	0	0	0	0	0	1	0	0	1	0	1
**Family Engraulidae**											
Amazonsprattus scintilla Roberts 1984	1	0	0	0	0	0	1	0	0	1	0
Anchovia surinamensis (Bleeker 1865)	1160	92	9	33	101	3	1193	193	12	1261	137
Anchoviella alleni (Myers 1940)	2	1	4	1	0	0	3	1	4	7	1
Anchoviella cf. *cayennensis* (Puyo 1946)	0	0	0	0	3	0	0	3	0	0	3
Anchoviella cf. *lepidentostole* (Fowler 1911)	47	1	49	89	10	0	136	11	49	97	99
Anchoviella guianensis (Eigenmann 1912)	2	3	0	12	0	0	14	3	0	5	12
Anchoviella juruasanga Loeb 2012	11	2	0	0	0	2	11	2	2	13	2
Anchoviella sp.	11	0	0	0	0	0	11	0	0	11	0
Lycengraulis batesii (Günther 1868)	1	0	97	0	2	1	1	2	98	98	3
Pterengraulis atherinoides (Linnaeus 1766)	0	0	10	0	0	8	0	0	18	10	8
**Family Pritigasteridae**											
Ilisha amazonica (Miranda Ribeiro 1920)	0	3	2	0	0	2	0	3	4	5	2
Pellona flavipinnis (Valenciennes 1837)	1	4	27	0	2	40	1	6	67	32	42
**Order Characiformes**											
**Family Erythrinidae**											
Hoplias malabaricus (Bloch 1794)	5	0	0	5	1	28	10	1	28	5	34
**Family Cynodontidae**											
Cynodon gibbus (Spix & Agassiz 1829)	0	1	0	0	0	15	0	1	15	1	15
Hydrolycus scomberoides (Cuvier 1819)	0	0	6	0	0	21	0	0	27	6	21
Rhaphiodon vulpinus Spix & Agassiz 1829	0	1	19	0	0	24	0	1	43	20	24
**Family Serrasalmidae**											
Catoprion mento (Cuvier 1819)	0	1	0	3	0	0	3	1	0	1	3
Colossoma macropomum (Cuvier 1816)	0	0	0	0	1	0	0	1	0	0	1
Metynnis hypsauchen (Müller & Troschel 1844)	6	1	0	1	0	0	7	1	0	7	1
Metynnis lippincottianus (Cope 1870)	0	0	0	0	1	0	0	1	0	0	1
Metynnis luna Cope 1878	7	40	0	1	1	3	8	41	3	47	5
Myloplus schomburgkii (Jardine 1841)	0	1	0	0	0	0	0	1	0	1	0
Myloplus sp.	0	0	0	1	1	0	1	1	0	0	2
Mylossoma aureum (Spix & Agassiz 1829)	0	0	3	0	0	29	0	0	32	3	29
Mylossoma duriventre (Cuvier 1818)	0	0	2	0	0	25	0	0	27	2	25
Pristobrycon striolatus (Steindachner 1908)	0	0	0	0	0	1	0	0	1	0	1
Pygocentrus nattereri Kner 1858	0	0	2	0	0	13	0	0	15	2	13
Pygopristis denticulata (Cuvier 1819)	1	0	0	2	0	1	3	0	1	1	3
Serrasalmus eigenmanni Norman 1929	1	0	1	0	0	2	1	0	3	2	2
Serrasalmus elongatus Kner 1858	0	0	0	0	0	1	0	0	1	0	1
Serrasalmus gouldingi Fink & Machado-Allison 1992	7	1	0	1	0	0	8	1	0	8	1
Serrasalmus maculatus Kner 1858	1	2	3	0	0	54	1	2	57	6	54
Serrasalmus manueli (Fernández-Yépez & Ramírez 1967)	0	0	0	1	0	0	1	0	0	0	1
Serrasalmus rhombeus (Linnaeus 1766)	1	0	1	0	1	14	1	1	15	2	15

Taxon	River Margin (RM)			Floodplain lake (FL)			RM & FL			RM	FL
	BW	CW	WW	BW	CW	WW	BW	CW	WW		
**Family Hemiodontidae**											
Anodus cf. *elongatus* Agassiz 1829	0	0	1	0	5	3	0	5	4	1	8
Argonectes robertsi Langeani 1999	0	6	0	6	4	0	6	10	0	6	10
Bivibranchia fowleri (Steindachner 1908)	711	224	2	18	46	0	729	270	2	937	64
Hemiodus atranalis (Fowler 1940)	0	0	0	0	6	0	0	6	0	0	6
Hemiodus gracilis Günther 1864	111	7	0	131	2	0	242	9	0	118	133
Hemiodus immaculatus Kner 1858	25	11	0	19	54	0	44	65	0	36	73
Hemiodus microlepis (Kner 1858)	1	6	1	2	72	2	3	78	3	8	76
Hemiodus unimaculatus (Bloch 1794)	5	9	0	9	36	0	14	45	0	14	45
Micromischodus sugillatus Roberts 1971	4	0	0	12	2	0	16	2	0	4	14
**Family Anostomidae**											
Anostomoides laticeps (Eigenmann 1912)	0	0	0	0	1	0	0	1	0	0	1
Laemolyta proxima (Garman 1890)	0	19	1	1	61	13	1	80	14	20	75
Laemolyta taeniata (Kner 1858)	0	1	0	1	0	0	1	1	0	1	1
Leporinus altipinnis Borodin 1929	3	0	0	9	1	0	12	1	0	3	10
Leporinus fasciatus (Bloch 1794)	1	14	0	0	11	0	1	25	0	15	11
Leporinus friderici (Bloch 1794)	0	0	0	1	0	2	1	0	2	0	3
Megaleporinus trifasciatus (Steindachner 1876)	0	1	1	0	0	1	0	1	2	2	1
Pseudanos gracilis (Kner 1858)	2	0	0	0	0	0	2	0	0	2	0
Pseudanos trimaculatus (Kner 1858)	0	1	0	0	0	0	0	1	0	1	0
Rhytiodus argenteofuscus Kner 1858	0	1	6	0	4	2	0	5	8	7	6
Rhytiodus microlepis Kner 1858	0	0	6	0	0	6	0	0	12	6	6
Schizodon fasciatus Spix & Agassiz 1829	1	4	8	0	3	57	1	7	65	13	60
Schizodon vittatus (Valenciennes 1850)	7	7	0	0	0	2	7	7	2	14	2
**Family Chilodontidae**											
Caenotropus labyrinthicus (Kner 1858)	1	3	0	0	8	0	1	11	0	4	8
**Family Curimatidae**											
Curimata ocellata Eigenmann & Eigenmann 1889	0	0	0	0	0	1	0	0	1	0	1
Curimata roseni Vari 1989	1	24	2	0	26	1	1	50	3	27	27
Curimata vittata (Kner 1858)	0	0	0	19	5	14	19	5	14	0	38
Curimatella alburnus (Müller & Troschel 1844)	0	0	0	0	0	102	0	0	102	0	102
Curimatella immaculata (Fernández-Yépez 1948)	149	0	0	1	1	0	150	1	0	149	2
Curimatella meyeri (Steindachner 1882)	111	0	1	0	0	92	111	0	93	112	92
Cyphocharax leucostictus (Eigenmann & Eigenmann	0	34	1	80	58	0	80	92	1	35	138
Cyphocharax plumbeus (Eigenmann & Eigenmann 1889)	0	0	6	0	0	27	0	0	33	6	27
Cyphocharax spiluropsis (Eigenmann & Eigenmann	0	0	0	0	0	73	0	0	73	0	73
Potamorhina altamazonica (Cope 1878)	0	0	1	0	0	3	0	0	4	1	3
Potamorhina latior (Spix & Agassiz 1829)	0	0	0	0	0	25	0	0	25	0	25
Potamorhina sp.	15	0	1	4	0	0	19	0	1	16	4
Pristigaster cayana Cuvier 1829	0	1	0	0	0	6	0	1	6	1	6
Psectrogaster amazonica Eigenmann & Eigenmann 1889	0	0	2	0	0	11	0	0	13	2	11
Psectrogaster rutiloides (Kner 1858	1	0	0	0	2	10	1	2	10	1	12
Steindachnerina bimaculata (Steindachner 1876)	0	2	1	0	12	486	0	14	487	3	498
**Family Prochilodontidae**											
Prochilodus nigricans Spix & Agassiz 1829	0	0	0	0	0	1	0	0	1	0	1
Semaprochilodus insignis (Jardine 1841)	0	0	0	0	1	0	0	1	0	0	1
Semaprochilodus taeniurus (Valenciennes 1821)	0	0	0	2	0	0	2	0	0	0	2
**Family Lebiasinidae**											
Copella arnoldi (Regan 1912)	0	0	0	0	0	2	0	0	2	0	2
Nannostomus digrammus (Fowler 1913)	0	0	0	40	0	0	40	0	0	0	40
Nannostomus eques Steindachner 1876	0	0	0	1	0	0	1	0	0	0	1
Pyrrhulina cf. *obermulleri* Myers 1926	0	0	0	0	0	2	0	0	2	0	2

Taxon	River Margin (RM)			Floodplain lake (FL)			RM & FL			RM	FL
	BW	CW	WW	BW	CW	WW	BW	CW	WW		
**Family Ctenoluciidae**											
Boulengerella cuvieri (Spix & Agassiz 1829)	12	14	1	0	1	2	12	15	3	27	3
Boulengerella lucius (Cuvier 1816)	2	0	0	1	0	0	3	0	0	2	1
Boulengerella maculata (Valenciennes 1850)	9	4	3	7	15	0	16	19	3	16	22
**Family Tripotheidae**											
Agoniates halecinus Müller & Troschel 1845	22	2	0	44	5	1	66	7	1	24	50
Triportheus albus Cope 1872	68	35	4	25	3	14	93	38	18	107	42
Triportheus auritus (Valenciennes 1850)	7	2	23	2	4	12	9	6	35	32	18
Triportheus cf. *angulatus* (Spix & Agassiz 1829)	1	2	35	0	3	155	1	5	190	38	158
**Family Gasteropelecidae**											
Thoracocharax stellatus (Kner 1858)	0	0	4	0	0	0	0	0	4	4	0
**Family Bryconidae**											
Brycon melanopterus (Cope 1872)	0	0	0	4	0	0	4	0	0	0	4
Brycon "pesu" sp. A	1	0	0	0	0	0	1	0	0	1	0
Brycon "pesu" sp. B	8	4	0	0	5	0	8	9	0	12	5
Chalceus epakros Zanata & Toledo-Piza 2004	0	13	0	0	14	0	0	27	0	13	14
Chalceus erythrurus (Cope 1870)	0	0	0	0	0	1	0	0	1	0	1
**Family Iguanodectidae**											
Bryconops alburnoides Kner 1858	47	19	0	14	16	3	61	35	3	66	33
Bryconops caudomaculatus (Günther 1864)	4	0	0	0	4	17	4	4	17	4	21
Bryconops cf. *melanurus* (Bloch 1794	0	0	0	111	0	0	111	0	0	0	111
Bryconops cf. *transitoria* Steindachner 1915	126	57	2	111	1011	0	237	1068	2	185	1122
Bryconops durbinae (Eigenmann 1908)	244	1	0	275	394	0	519	395	0	245	669
Iguanodectes polylepis Géry 199	0	0	0	3	2	2	3	2	2	0	7
**Family Acestrorhynchidae**											
Acestrorhynchus abbreviatus (Cope 1878)	1	0	3	0	0	80	1	0	83	4	80
Acestrorhynchus falcirostris (Cuvier 1819)	4	5	0	15	17	3	19	22	3	9	35
Acestrorhynchus microlepis (Jardine 1841)	120	54	0	17	42	1	137	96	1	174	60
Acestrorhynchus minimus Menezes 1969	16	0	0	37	73	0	53	73	0	16	110
Lonchogenys ilisha Myers 1927	11	0	0	0	0	0	11	0	0	11	0
**Family Characidae**											
Aphyocharax alburnus (Günther 1869)	0	0	4	0	0	6	0	0	10	4	6
Aphyocharax avary Fowler 1913	0	0	0	0	0	58	0	0	58	0	58
Aphyocharax nattereri (Steindachner 1882)	0	0	0	0	0	2	0	0	2	0	2
Aphyodite grammica Eigenmann 1912	0	0	0	0	134	298	0	134	298	0	432
Astyanax guianensis Eigenmann 1909	5	0	0	0	29	0	5	29	0	5	29
Charax sp.	0	0	0	0	0	24	0	0	24	0	24
Cheirodon micropterus Eigenmann 1907	0	0	0	0	7	75	0	7	75	0	82
Creagrutus cracentis Vari & Harold 2001	11	0	0	0	0	1	11	0	1	11	1
Ctenobrycon spilurus (Valenciennes 1850)	0	0	0	0	0	368	0	0	368	0	368
Hemigrammus analis Durbin 1909	0	1	0	0	7	0	0	8	0	1	7
Hemigrammus diagonicus Mendonça & Wosiacki 2011	0	0	0	0	0	485	0	0	485	0	485
Hemigrammus durbinae Ota, Lima & Pavanelli 2015	0	0	0	0	0	1	0	0	1	0	1
Hemigrammus hyanuary Durbin 1918	0	0	0	0	9	0	0	9	0	0	9
Hemigrammus levis Durbin 1908	37	1	0	105	223	0	142	224	0	38	328
Hemigrammus lunatus Durbin 1918	0	0	0	0	0	361	0	0	361	0	361
Hemigrammus sp.	0	0	0	0	0	1	0	0	1	0	1
Hemigrammus unilineatus (Gill 1858)	0	0	0	0	0	4	0	0	4	0	4
Hyphessobrycon copelandi Durbin 1908	0	0	0	0	0	4	0	0	4	0	4
Hyphessobrycon eques (Steindachner 1882)	0	0	0	0	0	114	0	0	114	0	114
Hyphessobrycon hasemani Fowler 1913	0	0	0	44	0	0	44	0	0	0	44
Jupiaba cf. *essequibensis* (Eigenmann 1909)	68	9	0	0	0	0	68	9	0	77	0

Taxon	River Margin (RM)			Floodplain lake (FL)			RM & FL			RM	FL
	BW	CW	WW	BW	CW	WW	BW	CW	WW		
Knodus heteresthes (Eigenmann 1908	90	520	62	0	20	0	90	540	62	672	20
Microschemobrycon melanotus (Eigenmann 1912)	1	0	0	0	0	0	1	0	0	1	0
Microschemobrycon sp.	0	1	0	0	652	0	0	653	0	1	652
Moenkhausia celibela Marinho & Langeani 2010	166	0	0	17	0	21	183	0	21	166	38
Moenkhausia ceros Eigenmann 1908	25	0	0	17	0	0	42	0	0	25	17
Moenkhausia cf. *jamesi* Eigenmann 1908	0	4	2	0	1	0	0	5	2	6	1
Moenkhausia grandisquamis (Müller & Troschel 1845)	0	1	0	0	3	0	0	4	0	1	3
Moenkhausia intermedia Eigenmann 1908	0	0	0	0	0	383	0	0	383	0	383
Moenkhausia lata Eigenmann 1908	86	62	10	0	2	0	86	64	10	158	2
Moenkhausia lepidura (Kner 1858)	15	8	4	422	30	33	437	38	37	27	485
Moenkhausia megalops (Eigenmann 1907)	0	2	0	0	0	0	0	2	0	2	0
Moenkhausia oligolepis (Günther 1864)	0	0	1	0	0	6	0	0	7	1	6
Moenkhausia sp.	0	0	0	0	0	4	0	0	4	0	4
Odontostilbe fugitiva Cope 1870	0	0	5	0	0	0	0	0	5	5	0
Phenacogaster cf. *retropinnus* Lucena & Malabarba	3	0	0	0	0	0	3	0	0	3	0
Phenacogaster pectinatus (Cope 1870)	4	0	0	0	0	0	4	0	0	4	0
Prionobrama filigera (Cope 1870)	0	0	12	0	0	679	0	0	691	12	679
Roeboides affinis (Günther 1868	42	0	1	0	0	11	42	0	12	43	11
Roeboides biserialis (Garman 1890)	3	0	1	0	0	3	3	0	4	4	3
Roeboides descalvadensis Fowler 1932	0	0	0	0	2	1526	0	2	1526	0	1528
Roeboides myersii Gill 1870	0	0	6	0	1	44	0	1	50	6	45
Stethaprion crenatum Eigenmann 1916	0	0	0	0	0	1	0	0	1	0	1
Tetragonopterus argenteus Cuvier 1816	0	0	2	0	0	2	0	0	4	2	2
Tetragonopterus chalceus Spix & Agassiz 1829	1	12	0	0	0	0	1	12	0	13	0
**Order Gymnotiformes**											
**Family Apteronotidae**											
Adontosternarchus balaenops (Cope, 1878)	0	0	1	0	0	0	0	0	1	1	0
Adontosternarchus clarkae Mago-Leccia, Lundberg, Baskin, 1985	0	0	2	0	0	0	0	0	2	2	0
Orthosternarchus tamandua (Boulenger, 1898)	0	0	1	0	0	0	0	0	1	1	0
Parapteronotus hasemani (Ellis, 1913)	0	0	2	0	0	5	0	0	7	2	5
Platyurosternarchus macrostoma (Günther, 1870)	0	0	3	0	0	0	0	0	3	3	0
Porotergus gimbeli Ellis, 1912	0	0	1	0	0	0	0	0	1	1	0
Sternarchella calhamazon Lundberg, Cox Fernandes, Campos-da-Paz, 2013	0	0	4	0	0	0	0	0	4	4	0
Sternarchella orthos Mago-Leccia, 1994	0	0	26	0	0	0	0	0	26	26	0
Sternarchella schotti (Steindachner, 1868)	0	0	0	0	0	1	0	0	1	0	1
Sternarchogiton nattereri (Steindachner, 1868)	0	0	12	0	0	0	0	0	12	12	0
Sternarchogiton porcinum Eigenmann, Allen, 1942	0	0	2	0	0	0	0	0	2	2	0
Sternarchogiton preto de Santana, Crampton, 2007	0	0	1	0	0	0	0	0	1	1	0
Sternarchorhamphus muelleri (Steindachner, 1881)	0	0	1	0	0	0	0	0	1	1	0
Sternarchorhynchus goeldii de Santana, Vari, 2010	0	0	1	0	0	0	0	0	1	1	0
Sternarchorhynchus retzeri de Santana, Vari, 2010	0	0	1	0	0	0	0	0	1	1	0
**Family Sternopygidae**											
Distocyclus conirostris (Eigenmann, Allen, 1942)	1	0	0	0	1	0	1	1	0	1	1
Eigenmannia limbata (Schreiner, Miranda Ribeiro,	0	0	1	0	1	150	0	1	151	1	151
Eigenmannia macrops (Boulenger, 1897)	0	0	3	0	0	0	0	0	3	3	0
Eigenmannia sp.	0	0	0	0	0	678	0	0	678	0	678
Rhabdolichops electrogrammus Lundberg, Mago-Leccia, 1986	0	0	3	0	0	0	0	0	3	3	0
Rhabdolichops lundbergi Correa, Crampton, Albert,	0	0	3	0	0	0	0	0	3	3	0
Rhabdolichops sp.	0	0	5	0	0	0	0	0	5	5	0
Rhabdolichops troscheli (Kaup, 1856)	0	1	0	0	0	0	0	1	0	1	0
Sternopygus macrurus (Bloch, Schneider, 1801)	0	0	1	0	0	25	0	0	26	1	25
**Family Hypopomidae**											
Brachyhypopomus pinnicaudatus (Hopkins, Comfort, Bastian, Bass, 1990)	0	0	0	0	0	1	0	0	1	0	1

Taxon	River Margin (RM)			Floodplain lake (FL)			RM & FL			RM	FL
	BW	CW	WW	BW	CW	WW	BW	CW	WW		
**Family Rhamphichthyidae**											
Rhamphichthys drepanium Triques, 1999	0	0	0	0	0	7	0	0	7	0	7
Rhamphichthys heleios Carvalho, Albert, 2015	0	0	1	0	0	1	0	0	2	1	1
Rhamphichthys lineatus Castelnau, 1855	0	0	0	0	0	2	0	0	2	0	2
Rhamphichthys marmoratus Castelnau 1855	0	0	0	0	1	1	0	1	1	0	2
Rhamphichthys rostratus (Linnaeus, 1766)	0	0	1	0	0	2	0	0	3	1	2
Steatogenys elegans (Steindachner, 1880)	0	0	46	0	0	0	0	0	46	46	0
**Order Siluriformes**											
**Family Trichomycteridae**											
Vandellia cirrhosa Valenciennes 1846	0	2	1	0	0	0	0	2	1	3	0
**Family Callichthyidae**											
Corydoras hastatus Eigenmann & Eigenmann 1888	0	0	1	0	0	0	0	0	1	1	0
Hoplosternum littorale (Hancock 1828)	0	0	0	0	0	48	0	0	48	0	48
**Family Loricariidae**											
Ancistrus cf. *hoplogenys* (Günther 1864)	0	0	0	0	4	0	0	4	0	0	4
Dekeyseria scaphirhynchus (Kner 1854)	0	0	0	1	0	0	1	0	0	0	1
Farlowella amazonum (Günther 1864)	0	0	0	0	0	11	0	0	11	0	11
Hemiodontichthys acipenserinus (Kner 1853)	0	0	1	0	0	5	0	0	6	1	5
Hypoptopoma incognitum Aquino & Schaefer 2010	0	0	10	0	4	6	0	4	16	10	10
Hypostomus cf. *plecostomus* (Linnaeus 1758)	0	0	0	0	1	4	0	1	4	0	5
Limatulichthys griseus (Eigenmann 1909)	25	30	1	5	99	1	30	129	2	56	105
Loricaria cf. *cataphracta* Linnaeus 1758	0	0	13	0	1	0	0	1	13	13	1
Loricariichthys acutus (Valenciennes 1840)	0	1	3	0	7	80	0	8	83	4	87
Loricariichthys nudirostris (Kner 1853)	0	0	1	0	3	6	0	3	7	1	9
Loricariichthys platymetopon Isbrücker & Nijssen 1979	0	0	0	0	2	27	0	2	27	0	29
Peckoltia vittata (Steindachner 1881)	5	5	1	1	6	1	6	11	2	11	8
Pseudohemiodon sp.	0	0	1	0	0	0	0	0	1	1	0
Pterygoplichthys gibbiceps (Kner 1854)	0	0	0	0	0	1	0	0	1	0	1
Pterygoplichthys pardalis (Castelnau 1855)	0	0	0	0	1	3	0	1	3	0	4
Pterygoplichthys punctatus (Kner 1854)	0	0	0	0	0	4	0	0	4	0	4
Rineloricaria sp.	0	0	0	0	0	4	0	0	4	0	4
Squaliforma horrida (Kner 1854)	0	0	1	0	0	0	0	0	1	1	0
Sturisoma cf. *rostratum* (Spix & Agassiz 1829)	0	0	3	0	1	8	0	1	11	3	9
**Family Cetopsidae**											
Cetopsis coecutiens (Lichtenstein 1819)	0	6	0	0	0	0	0	6	0	6	0
**Family Auchenipteridae**											
Ageneiosus inermis (Linnaeus 1766)	0	0	1	0	0	6	0	0	7	1	6
Ageneiosus sp.	4	2	12	2	0	30	6	2	42	18	32
Ageneiosus ucayalensis Castelnau 1855	0	2	6	0	0	15	0	2	21	8	15
Auchenipterichthys longimanus (Günther 1864)	2	8	0	25	18	0	27	26	0	10	43
Auchenipterus ambyiacus Fowler 1915	0	0	6	0	0	6	0	0	12	6	6
Auchenipterus cf. *nuchalis* (Spix & Agassiz 1829)	1	2	6	0	2	0	1	4	6	9	2
Centromochlus existimatus Mees 1974	0	4	28	0	0	0	0	4	28	32	0
Centromochlus heckelii (De Filippi 1853)	0	0	6	0	0	1	0	0	7	6	1
Tatia intermedia (Steindachner 1877)	0	1	0	0	0	0	0	1	0	1	0
Tatia melanoleuca Vari & Calegari 2014	0	21	0	0	2	0	0	23	0	21	2
Tatia nigra Sarmento-Soares & Martins-Pinheiro 2008	0	0	0	3	0	0	3	0	0	0	3
Tatia sp.	0	2	2	0	0	0	0	2	2	4	0
Trachelyichthys decaradiatus Mees 1974	0	0	0	17	0	0	17	0	0	0	17
Trachelyopterus galeatus (Linnaeus 1766)	2	0	0	10	112	233	12	112	233	2	355
Tympanopleura atronasus (Eigenmann & Eigenmann	0	0	1	0	0	9	0	0	10	1	9
Tympanopleura longipinna Walsh, Ribeiro & Rapp Py-Daniel 2015	0	0	4	0	0	1	0	0	5	4	1
Tympanopleura rondoni (Miranda-Ribeiro 1914)	0	0	3	0	0	13	0	0	16	3	13

Taxon	River Margin (RM)			Floodplain lake (FL)			RM & FL			RM	FL
	BW	CW	WW	BW	CW	WW	BW	CW	WW		
**Family Doradidae**											
Agamyxis pectinifrons (Cope 1870)	0	0	0	0	0	2	0	0	2	0	2
Anadoras grypus (Cope 1872)	0	0	0	0	0	88	0	0	88	0	88
Anduzedoras oxyrhynchus (Valenciennes 1821)	0	0	1	0	0	0	0	0	1	1	0
Astrodoras sp.	0	0	0	0	0	1	0	0	1	0	1
Hassar orestis (Steindachner 1875)	20	35	1	0	14	0	20	49	1	56	14
Hemidoras morei (Steindachner 1881)	0	0	2	0	0	0	0	0	2	2	0
Nemadoras elongatus (Boulenger 1898)	0	0	1	0	0	0	0	0	1	1	0
Opsodoras morei (Steindachner 1881)	0	0	0	2	0	0	2	0	0	0	2
Ossancora asterophysa Birindelli & Sabaj Pérez 2011	0	0	0	0	0	23	0	0	23	0	23
Oxydoras niger (Valenciennes 1821)	0	0	0	0	0	7	0	0	7	0	7
Platydoras armatulus (Valenciennes 1840)	0	0	0	0	0	2	0	0	2	0	2
Pterodoras sp.	0	0	1	0	0	3	0	0	4	1	3
Scorpiodoras heckelii (Kner 1855)	8	0	0	4	0	0	12	0	0	8	4
Trachydoras brevis (Kner 1853)	0	0	0	0	0	1	0	0	1	0	1
Trachydoras nattereri (Steindachner 1881)	0	0	1	0	0	69	0	0	70	1	69
Trachydoras sp.	0	0	2	0	0	4	0	0	6	2	4
Trachydoras steindachneri (Perugia 1897)	0	0	5	0	0	1	0	0	6	5	1
**Family Heptapteridae**											
Pimelodella cf. *cristata* (Müller & Troschel 1849)	11	3	0	0	6	0	11	9	0	14	6
Pimelodella cf. *laticeps* Eigenmann 1917	0	0	2	0	5	230	0	5	232	2	235
Pimelodella sp. A	0	0	13	0	0	361	0	0	374	13	361
Pimelodella sp. B	0	0	2	0	0	0	0	0	2	2	0
**Family Pimelodidae**											
Brachyplatystoma vaillantii (Valenciennes 1840)	0	0	1	0	0	1	0	0	2	1	1
Calophysus macropterus (Lichtenstein 1819)	0	0	13	0	0	9	0	0	22	13	9
Hemisorubim platyrhynchos (Valenciennes 1840)	0	0	0	0	0	1	0	0	1	0	1
Hypophthalmus edentatus Spix and Agassiz 1829	0	0	0	0	0	2	0	0	2	0	2
Hypophthalmus fimbriatus Kner 1858	0	1	1	0	3	0	0	4	1	2	3
Hypophthalmus marginatus Valenciennes 1840	0	0	19	0	0	0	0	0	19	19	0
Pimelodus blochii Valenciennes 1840	11	0	18	0	0	54	11	0	72	29	54
Pimelodus tetramerus Ribeiro & Lucena 2006	21	27	2	0	44	6	21	71	8	50	50
Platysilurus mucosus (Vaillant 1880)	0	0	2	0	0	0	0	0	2	2	0
Propimelodus cf. *eigenmanni* (Van der Stigchel 1946)	0	0	52	0	0	0	0	0	52	52	0
Pseudoplatystoma fasciatum (Linnaeus 1766)	0	0	0	0	0	3	0	0	3	0	3
Pseudoplatystoma punctifer (Castelnau 1855)	0	0	0	0	0	2	0	0	2	0	2
Pseudoplatystoma tigrinum (Valenciennes 1840)	0	0	0	0	0	7	0	0	7	0	7
Sorubim elongatus Littmann, Burr, Schmidt & Isern	0	0	0	0	0	1	0	0	1	0	1
Sorubim lima (Bloch & Schneider 1801)	0	0	5	0	1	9	0	1	14	5	10
Sorubim maniradii Littmann, Burr & Buitrago-Suarez	0	2	0	0	0	2	0	2	2	2	2
Sorubimichthys planiceps (Spix & Agassiz 1829)	0	1	0	0	1	1	0	2	1	1	2
**Order Gobiiformes**											
**Family Eleotridae**											
Leptophilypnion pusillus Roberts 2013	0	0	0	1	0	0	1	0	0	0	1
Microphilypnus ternetzi Myers 1927	0	0	0	123	4	0	123	4	0	0	127
**Order Synbranchiformes**											
**Family Synbranchidae**											
Synbranchus madeirae Rosen & Rumney 1972	0	0	1	0	0	0	0	0	1	1	0
**Order Pleuronectiformes**											
**Family Achiridae**											
Hypoclinemus mentalis (Günther 1862)	0	3	1	0	27	0	0	30	1	4	27

Taxon	River Margin (RM)			Floodplain lake (FL)			RM & FL			RM	FL
	BW	CW	WW	BW	CW	WW	BW	CW	WW		
**Order Cichliformes**											
**Family Cichlidae**											
Acarichthys heckelii (Müller & Troschel 1849)	6	13	0	223	84	3	229	97	3	19	310
Acaronia nassa (Heckel 1840)	0	0	0	5	0	4	5	0	4	0	9
Apistogramma cf. *gephyra* Kullander 1980	0	0	0	1	0	0	1	0	0	0	1
Apistogramma pertensis (Haseman 1911)	0	0	0	3	6	0	3	6	0	0	9
Apistogramma regani Kullander 198	0	0	0	3	0	58	3	0	58	0	61
Astronotus ocellatus (Agassiz 1831)	0	0	0	0	0	2	0	0	2	0	2
Biotoecus opercularis (Steindachner 1875)	0	0	0	156	26	0	156	26	0	0	182
Chaetobranchopsis orbicularis (Steindachner 1875)	0	0	0	0	0	14	0	0	14	0	14
Cichla monoculus Spix & Agassiz 1831	0	0	0	0	20	12	0	20	12	0	32
Cichla pinima Kullander & Ferreira 2006	23	28	0	7	25	0	30	53	0	51	32
Cichlasoma amazonarum Kullander 1983	0	0	0	0	0	1	0	0	1	0	1
Crenicichla acutirostris Günther 1862	1	0	0	1	1	0	2	1	0	1	2
Crenicichla cf. *inpa* Ploeg 1991	0	0	0	0	0	4	0	0	4	0	4
Crenicichla johanna Heckel 1840	0	0	0	0	1	0	0	1	0	0	1
Crenicichla macrophthalma Heckel 1840	2	1	0	0	0	0	2	1	0	3	0
Crenicichla marmorata Pellegrin 1904	0	0	0	1	0	0	1	0	0	0	1
Crenicichla multispinosa (Pellegrin 1903)	0	0	0	0	1	0	0	1	0	0	1
Crenicichla regani Ploeg 1989	0	1	0	2	0	0	2	1	0	1	2
Crenicichla reticulata (Heckel 1840)	0	4	2	0	0	3	0	4	5	6	3
Crenicichla sp.	0	0	0	3	2	0	3	2	0	0	5
Geophagus altifrons Heckel 1840	188	57	16	30	224	5	218	281	21	261	259
Geophagus sp.	0	2	0	2	102	12	2	104	12	2	116
Hypselecara temporalis (Günther 1862)	0	0	0	1	0	2	1	0	2	0	3
Laetacara curviceps (Ahl 1923)	0	0	0	26	2	4	26	2	4	0	32
Mesonauta festivus (Heckel 1840)	21	0	0	10	0	199	31	0	199	21	209
Mesonauta insignis (Heckel 1840)	0	0	0	16	1	95	16	1	95	0	112
Pterophyllum scalare (Schultze 1823)	0	0	0	0	0	33	0	0	33	0	33
Satanoperca acuticeps (Heckel 1840)	1	0	0	12	4	1	13	4	1	1	17
Satanoperca daemon (Heckel 1840)	0	0	0	5	0	0	5	0	0	0	5
Satanoperca jurupari (Heckel 1840)	0	0	1	0	3	58	0	3	59	1	61
Taeniacara candidi Myers 1935	0	0	0	0	2	0	0	2	0	0	2
**Order Cyprinodontiformes**											
**Family Fluviphylacidae**											
Fluviphylax cf. *simplex* Costa 1996	0	0	0	10	0	0	10	0	0	0	10
**Family Poeciliidae**											
Pamphorichthys scalpridens (Garman 1895)	6	0	0	23	0	15	29	0	15	6	38
**Order Beloniformes**											
**Family Hemiramphidae**											
Hyporhamphus brederi (Fernández-Yépez 1948)	1	1	2	0	0	0	1	1	2	4	0
**Family Belonidae**											
Potamorrhaphis guianensis (Jardine 1843)	0	0	1	0	0	5	0	0	6	1	5
Pseudotylosurus angusticeps (Günther 1866	0	1	0	0	0	0	0	1	0	1	0
Pseudotylosurus microps (Günther 1866)	0	7	6	1	0	0	1	7	6	13	1
**Order Tetraodontiformes**											
**Family Tetraodontidae**											
Colomesus asellus (Müller & Troschel 1849)	17	50	12	0	1	0	17	51	12	79	1
**Order Perciformes**											
**Family Sciaenidae**											
Pachypops fourcroi (Lacepède 1802)	26	33	13	0	5	2	26	38	15	72	7
Pachypops pigmaeus Casatti 2002	0	2	0	0	0	0	0	2	0	2	0
Plagioscion auratus (Castelnau 1855)	0	3	4	0	0	27	0	3	31	7	27
Plagioscion squamosissimus (Heckel 1840)	21	13	12	7	2	77	28	15	89	46	86
**Order Ceratodontiformes**											
**Family Lepidosirenidae**											
Lepidosiren paradoxa Fitzinger 1837	0	0	0	0	0	1	0	0	1	0	1

[1] Taxonomy follows: Eschmeyer WN, Van de Laan, R., Fricke R. 2019. Eschmeyer's catalog of fishes. http://researcharchive.calacademy.org/research/ichthyology/catalog/fishcatmain.asp (Electronic version: accessed 01 October 2019); Fricke R, Eschmeyer WN, Fong JD. 2019. Species by family/sub-family In Eschmeyer's Catalog of fishes. http://researcharchive.calacademy.org/research/ichthyology/catalog/SpeciesByFamily.asp (Electronic version accessed 01 October 2019).

Table S4. Permutational multivariate analysis of variance (PERMANOVA) comparisons of fish assemblage structure between blackwater (BW), clearwater (CW), and whitewater (WW) systems of the Santarém region. All comparisons are significant at $\alpha = 0.001$. DF = degrees of freedom.

Comparison	River Margins (RM)			Floodplain lakes (FL)			Pooled RM and FL		
	Pseudo *F*	R	DF	Pseudo *F*	R	DF	Pseudo *F*	R	DF
Global (BW-CW=WW)	4.5	0.47	2, 33	5.0	0.48	2, 34	7.4	0.41	2, 68
BW-CW	2.4	0.31	1, 22	2.6	0.32	1, 22	3.8	0.27	1, 46
BW-WW	5.0	0.44	1, 21	6.2	0.47	1, 22	8.5	0.4	1, 45
CW-WW	5.9	0.47	1, 21	6.2	0.47	1, 22	9.8	0.42	1, 45

Table S5. Results of generalized linear mixed models (GLMMs) for the effect of water type (BW = blackwater, CW = clearwater, WW = whitewater) and habitat type (river margin, floodplain lake) on fish biomass, abundance, and richness. For reach response variable we repeat the GLMM with the exclusion/inclusion of outlier sample events, and with the following data filtering procedures: For *biomass*: exclusion of large individuals (those with a mass exceeding 3 standard deviations of the mean fish mass for all our combined samples). For abundance and richness: exclusion of rare species (species represented by < 4 individuals in all our combined samples), exclusion of dominant species (the top 10% most numerically abundant species, in all our combined samples), exclusion of both rare and dominant species. Results are divided into: a. Type-3 Wald's test. b. Number of fish and sample event numbers for each water type/habitat combination. c. Marginal and conditional R^2 values. d. Tukey post-hoc comparisons of disparities between all water type/habitat combinations. Here only significant disparities at α = 0.05 are shown. e. Least square means and 95% confidence intervals. f. Original means.

**a.**				Wald's tests (bold = significant at alpha = 0.05)								
				Water type (WT)			Habitat type (HT)			WT:HT interaction		
Response	Outlier sample events	Data filtering	Figure	Chi-sq	d.f	*p*	Chi-sq	d.f.	*p*	Chi-sq	d.f.	*p*
Biomass	Excluded	None	Fig. 5b	57.5	2	**3.3E-13**	3.321	1	0.07	28.3	2	**7.0E-07**
Biomass	Included	None	-	21.1	2	**2.6E-05**	1.143	1	0.28	10.9	2	**4.3E-03**
Biomass	Excluded	Large individuals removed	-	29.9	2	**3.2E-07**	0.660	1	0.41	23.8	2	**6.6E-06**
Abundance	Excluded	None	Fig. 5c	22.4	2	**1.4E-05**	0.003	1	0.96	20.2	2	**2.7E-07**
Abundance	Included	None	-	9.9	2	**7.3E-03**	0.221	1	0.64	18.4	2	**1.0E-04**
Abundance	Excluded	Rare species excluded	-	2.3	2	**1.2E-05**	0.001	1	0.97	3.2	2	**1.1E-07**
Abundance	Excluded	Dominant species excluded	-	26.2	2	**2.0E-06**	0.415	1	0.52	20.8	2	**3.1E-05**
Abundance	Excluded	Rare and dominant species excluded	-	36.4	2	**1.3E-08**	0.792	1	0.37	28.7	2	**5.9E-07**
Richness	Excluded	None	Fig. 5a	32.1	2	**1.1E-07**	1.130	1	0.29	14.9	2	**5.9E-04**
Richness	Included	None	-	23.3	2	**8.6E-06**	0.850	1	0.36	11.0	2	**4.0E-03**
Richness	Excluded	Rare species excluded	-	43.1	2	**4.4E-10**	2.387	1	0.12	23.4	2	**8.4E-06**
Richness	Excluded	Dominant species excluded	-	40.4	2	**1.7E-09**	0.718	1	0.40	11.0	2	**4.2E-03**
Richness	Excluded	Rare and dominant species excluded	-	48.2	2	**3.4E-11**	1.217	1	0.27	16.2	2	**3.0E-04**

**b.**			n. fish / n. sample events							
Response	Outlier sample events	Data filtering	n fish	BW RM	BW FL	CW RM	CW FL	WW RM	WW FL	TOTAL
Biomass	Excluded	None	20070	12	11	12	10	11	12	68
Biomass	Included	None	22398	12	12	12	12	11	12	71
Biomass	Excluded	Large individuals removed	18449	11	11	11	10	10	12	65
Abundance	Excluded	None	13904	11	12	12	10	9	11	65
Abundance	Included	None	22398	12	12	12	12	11	12	71
Abundance	Excluded	Rare species excluded	13772	11	12	12	10	9	11	65
Abundance	Excluded	Dominant species excluded	5192	12	11	10	11	9	12	65
Abundance	Excluded	Rare and dominant species excluded	5063	12	11	10	11	9	12	65
Richness	Excluded	None	21344	12	12	10	12	10	12	68
Richness	Included	None	22398	12	12	12	12	11	12	71
Richness	Excluded	Rare species excluded	21197	12	12	10	12	10	12	68
Richness	Excluded	Dominant species excluded	5879	12	11	10	11	9	12	65
Richness	Excluded	Rare and dominant species excluded	5881	12	11	11	12	10	12	68

**c.**			R^2 values	
Response	Outlier sample events	Data filtering	Marginal R^2	Conditional R^2
Biomass	Excluded	None	0.429	0.573
Biomass	Included	None	0.284	0.489
Biomass	Excluded	Large individuals removed	0.428	0.618
Abundance	Excluded	None	0.136	0.803
Abundance	Included	None	0.085	0.796
Abundance	Excluded	Rare species excluded	0.141	0.807
Abundance	Excluded	Dominant species excluded	0.22	0.73
Abundance	Excluded	Rare and dominant species excluded	0.224	0.734
Richness	Excluded	None	0.216	0.681
Richness	Included	None	0.188	0.709
Richness	Excluded	Rare species excluded	0.222	0.673
Richness	Excluded	Dominant species excluded	0.293	0.613
Richness	Excluded	Rare and dominant species excluded	0.292	0.6

**d.**			Tukey post-hoc comparisons (bold = significant at alpha = 0.05)					
Response	Outlier sample events	Data filtering	BWF-BWR	*p*	BWF-CWF	*p*	BWF-CWR	*p*
Biomass	Excluded	None	BWF~BWR	0.4512	BWF~CWF	1	BWF~CWR	0.9459
Biomass	Included	None	BWF~BWR	0.8937	BWF~CWF	0.8462	BWF~CWR	0.9988
Biomass	Excluded	Large individuals removed	BWF~BWR	0.9654	BWF~CWF	0.9493	BWF~CWR	0.9332
Abundance	Excluded	None	BWF~BWR	1	BWF~CWF	0.3768	BWF~CWR	0.9961
Abundance	Included	None	BWF~BWR	0.9972	BWF~CWF	0.9998	BWF~CWR	1
Abundance	Excluded	Rare species excluded	BWF~BWR	1	BWF~CWF	0.35	BWF~CWR	0.9965
Abundance	Excluded	Dominant species excluded	BWF~BWR	0.9656	BWF~CWF	0.9999	BWF~CWR	0.9953
Abundance	Excluded	Rare and dominant species excluded	BWF~BWR	0.949	BWF~CWF	0.9999	BWF~CWR	0.9968
Richness	Excluded	None	BWF~BWR	0.8961	BWF~CWF	0.8951	BWF~CWR	0.9096
Richness	Included	None	BWF~BWR	0.9411	BWF~CWF	0.9414	BWF~CWR	0.7567
Richness	Excluded	Rare species excluded	BWF~BWR	0.6349	BWF~CWF	0.6984	BWF~CWR	0.7428
Richness	Excluded	Dominant species excluded	BWF~BWR	0.9584	BWF~CWF	0.8748	BWF~CWR	0.9234
Richness	Excluded	Rare and dominant species excluded	BWF~BWR	0.8801	BWF~CWF	0.7847	BWF~CWR	0.8079

BWF-WWF	*p*	BWF-WWR	*p*	BWR-CWF	*p*	BWR-CWR	*p*	BWR-WWF	*p*	BWR-WWR	*p*
BWF<WWF	**<.0001**	BWF~WWR	0.7118	BWR~CWF	0.5677	BWR~CWR	0.9309	BWR<WWF	**<.0001**	BWR~WWR	0.9991
BWF<WWF	**0.0001**	BWF~WWR	0.9643	BWR~CWF	1	BWR~CWR	0.9847	BWR<WWF	**0.0092**	BWR~WWR	0.9999
BWF<WWF	**<.0001**	BWF~WWR	0.9974	BWR~CWF	1	BWR~CWR	1	BWR<WWF	**0.0003**	BWR~WWR	0.8017
BWF<WWF	**0.0309**	BWF>WWR	**0.0173**	BWR~CWF	0.435	BWR~CWR	0.9979	BWR<WWF	**0.0326**	BWR>WWR	**0.0238**
BWF<WWF	**0.0484**	BWF~WWR	0.1668	BWR~CWF	1	BWR~CWR	0.9892	BWR~WWF	0.1466	BWR~WWR	0.0539
BWF<WWF	**0.0319**	BWF>WWR	**0.0096**	BWR~CWF	0.3922	BWR~CWR	0.9976	BWR<WWF	**0.0363**	BWR>WWR	**0.0124**
BWF<WWF	**<.0001**	BWF~WWR	0.8117	BWR~CWF	0.8962	BWR~CWR	0.7857	BWR<WWF	**0.0002**	BWR~WWR	0.3254
BWF<WWF	**<.0001**	BWF~WWR	0.721	BWR~CWF	0.8673	BWR~CWR	0.7678	BWR<WWF	**0.0003**	BWR~WWR	0.208
BWF<WWF	**<.0001**	BWF~WWR	0.9454	BWR~CWF	1	BWR~CWR	1	BWR<WWF	**0.0003**	BWR~WWR	1
BWF<WWF	**0.0001**	BWF~WWR	0.9076	BWR~CWF	1	BWR~CWR	0.9981	BWR<WWF	**0.004**	BWR~WWR	1
BWF<WWF	**<.0001**	BWF~WWR	0.9664	BWR~CWF	1	BWR~CWR	1	BWR<WWF	**<.0001**	BWR~WWR	0.986
BWF<WWF	**<.0001**	BWF~WWR	0.3769	BWR~CWF	0.9998	BWR~CWR	1	BWR<WWF	**<.0001**	BWR~WWR	0.8648
BWF<WWF	**<.0001**	BWF~WWR	0.4704	BWR~CWF	1	BWR~CWR	1	BWR<WWF	**<.0001**	BWR~WWR	0.9741

CWF-CWR	*p*	CWF-WWF	*p*	CWF-WWR	*p*	CWR-WWF	*p*	CWR-WWR	*p*	WWR-WWF	*p*
CWF~CWR	0.9775	CWF<WWF	**<.0001**	CWF~WWR	0.8053	CWR<WWF	**<.0001**	CWR~WWR	0.9933	WWR<WWF	**<.0001**
CWF~CWR	0.9657	CWF<WWF	**0.0236**	CWF~WWR	0.999	CWR<WWF	**0.0007**	CWR~WWR	0.998	WWR<WWF	**0.0056**
CWF~CWR	1	CWF<WWF	**0.0004**	CWF~WWR	0.7734	CWR<WWF	**0.0005**	CWR~WWR	0.7323	WWR<WWF	**<.0001**
CWF~CWR	0.6852	CWF<WWF	**<.0001**	CWF~WWR	0.7795	CWR<WWF	**0.0081**	CWR~WWR	0.0647	WWR<WWF	**<.0001**
CWF~CWR	0.9983	CWF~WWF	0.1234	CWF~WWR	0.1201	CWR<WWF	**0.0372**	CWR~WWR	0.2401	WWR<WWF	**<.0001**
CWF~CWR	0.6491	CWF<WWF	**<.0001**	CWF~WWR	0.7006	CWR<WWF	**0.0088**	CWR>WWR	**0.0379**	WWR<WWF	**<.0001**
CWF~CWR	0.9997	CWF<WWF	**<.0001**	CWF~WWR	0.9058	CWR<WWF	**<.0001**	CWR~WWR	0.9775	WWR<WWF	**<.0001**
CWF~CWR	0.9999	CWF<WWF	**<.0001**	CWF~WWR	0.8348	CWR<WWF	**<.0001**	CWR~WWR	0.9383	WWR<WWF	**<.0001**
CWF~CWR	1	CWF<WWF	**0.0003**	CWF~WWR	1	CWR<WWF	**0.0011**	CWR~WWR	1	WWR<WWF	**0.0006**
CWF~CWR	0.998	CWF<WWF	**0.004**	CWF~WWR	1	CWR<WWF	**0.0191**	CWR~WWR	0.9997	WWR<WWF	**0.0097**
CWF~CWR	1	CWF<WWF	**<.0001**	CWF~WWR	0.9932	CWR<WWF	**0.0002**	CWR~WWR	0.9939	WWR<WWF	**<.0001**
CWF~CWR	1	CWF<WWF	**<.0001**	CWF~WWR	0.9505	CWR<WWF	**0.0002**	CWR~WWR	0.9562	WWR<WWF	**0.0038**
CWF~CWR	1	CWF<WWF	**<.0001**	CWF~WWR	0.9931	CWR<WWF	**<.0001**	CWR~WWR	0.994	WWR<WWF	**0.0002**

e.

			Least square (ls) means and lowe (L) and upper (U) 95% confidence intervals																	
			BW RM			BW FL			CW RM			CW FL			WW RM			WW FL		
Response	Outlier sample events	Data filtering	ls mean	L 95%	U 95%	ls mean	L 95%	U 95%	ls mean	L 95%	U 95%	ls mean	L 95%	U 95%	ls mean	L 95%	U 95%	ls mean	L 95%	U 95%
Biomass	Excluded	None	8.04	7.52	8.57	7.6	7.07	8.14	7.82	7.29	8.34	7.64	7.09	8.18	7.95	7.42	8.49	9.21	8.68	9.73
Biomass	Included	None	8.03	7.4	8.67	7.67	7.04	8.31	7.81	7.17	8.44	8.09	7.43	8.75	7.96	7.31	8.61	9.18	8.55	9.82
Biomass	Excluded	Large individuals removed	7.35	6.7	8.01	7.11	6.46	7.75	7.39	6.74	8.04	7.37	6.72	8.03	6.97	6.31	7.63	8.62	7.98	9.26
Abundance	Excluded	None	4.74	3.74	5.73	4.75	3.76	5.74	4.62	3.63	5.61	4.21	3.2	5.22	3.8	2.78	4.83	5.54	4.55	6.54
Abundance	Included	None	4.9	3.74	6.05	4.75	3.6	5.9	4.7	3.55	5.86	4.84	3.68	6	4.02	2.86	5.18	5.62	4.47	6.77
Abundance	Excluded	Rare species excluded	4.73	3.73	5.73	4.73	3.74	5.73	4.61	3.61	5.6	4.18	3.17	5.2	3.74	2.71	4.77	5.53	4.53	6.53
Abundance	Excluded	Dominant species excluded	4	3.35	4.66	3.82	3.15	4.48	3.69	3.02	4.37	3.76	3.1	4.43	3.5	2.81	4.19	4.94	4.28	5.59
Abundance	Excluded	Rare and dominant species excluded	3.99	3.33	4.65	3.79	3.12	4.45	3.67	2.99	4.35	3.73	3.07	4.4	3.43	2.74	4.12	4.91	4.25	5.56
Richness	Excluded	None	2.92	2.49	3.34	2.73	2.3	3.16	2.92	2.48	3.36	2.92	2.49	3.34	2.9	2.46	3.34	3.63	3.22	4.05
Richness	Included	None	2.91	2.45	3.37	2.72	2.25	3.19	3	2.53	3.46	2.91	2.45	3.37	2.94	2.46	3.41	3.62	3.17	4.08
Richness	Excluded	Rare species excluded	2.88	2.49	3.27	2.64	2.24	3.04	2.87	2.46	3.28	2.86	2.47	3.26	2.77	2.36	3.18	3.54	3.16	3.92
Richness	Excluded	Dominant species excluded	2.62	2.25	3	2.47	2.08	2.85	2.66	2.26	3.07	2.67	2.3	3.05	2.84	2.45	3.23	3.49	3.13	3.85
Richness	Excluded	Rare and dominant species excluded	2.57	2.23	2.92	2.39	2.03	2.75	2.61	2.25	2.96	2.61	2.26	2.95	2.7	2.34	3.06	3.38	3.05	3.71

f.

Response	Outlier sample events	Data filtering	Original means						
			unit	BW RM	BW FL	CW RM	CW FL	WW RM	WW FL
Biomass	Excluded	None	grams	3813.9	1973.4	2609.4	2363.1	3177.6	11378.6
Biomass	Included	None	grams	3813.9	2333.6	2609.4	4409.3	3177.6	11378.6
Biomass	Excluded	Large individuals removed	grams	1950.3	1194.7	1553.8	1860.1	1094.9	7737.4
Abundance	Excluded	None	n	223.3	209.9	144.9	97.4	38.2	533.8
Abundance	Included	None	n	341.5	209.9	144.9	347.2	82.8	747.1
Abundance	Excluded	Rare species excluded	n	222.1	208.2	143.5	96.0	36.1	529.5
Abundance	Excluded	Dominant species excluded	n	78.5	54.5	35.9	58.1	31.9	197.1
Abundance	Excluded	Rare and dominant species excluded	n	77.3	52.8	35.0	56.8	29.8	192.6
Richness	Excluded	None	n	20.6	16.3	17.5	21.2	19.5	43.9
Richness	Included	None	n	20.6	16.3	21.1	21.2	22.8	43.9
Richness	Excluded	Rare species excluded	n	19.7	14.8	16.7	20.0	16.9	39.7
Richness	Excluded	Dominant species excluded	n	14.9	12.3	14.1	16.0	17.9	36.5
Richness	Excluded	Rare and dominant species excluded	n	14.0	11.5	13.5	14.8	15.3	32.3
