# Supplementary material for: Biogeochemical water type influences community composition, species richness, and biomass in megadiverse Amazonian fish assemblages
Source: Sci Rep. 2020 Sep 18;10:15349. doi: 10.1038/s41598-020-72349-0 (PMC7501869; doi:10.1038/s41598-020-72349-0)

Online Resource

Supplementary Figures

Biogeochemical water type influences community composition, species richness, and biomass in megadiverse Amazonian fish assemblages

Juan David Bogotá-Gregory, Flávio C. T. Lima, Sandra B. Correa, Cárlison Silva-Oliveira, David G. Jenkins, Frank R. Ribeiro, Nathan R. Lovejoy, Roberto E. Reis, William G. R. Crampton*

* Corresponding author: Department of Biology, University of Central Florida, 4100 Libra Dr., 32816 Orlando, FL, USA. Email: crampton@ucf.edu

Fig. S1. Non-metric multidimensional scaling (NMDS) ordination for fish assemblage structure. Here all species with < 4 individuals are removed (and outlier sample events removed). Polygons represent convex hulls for the three water types. Environmental vectors significant at $\alpha = 0.05$ are displayed with R^2 value in parentheses. DO = dissolved oxygen, EC = electrical conductivity, TB = turbidity.

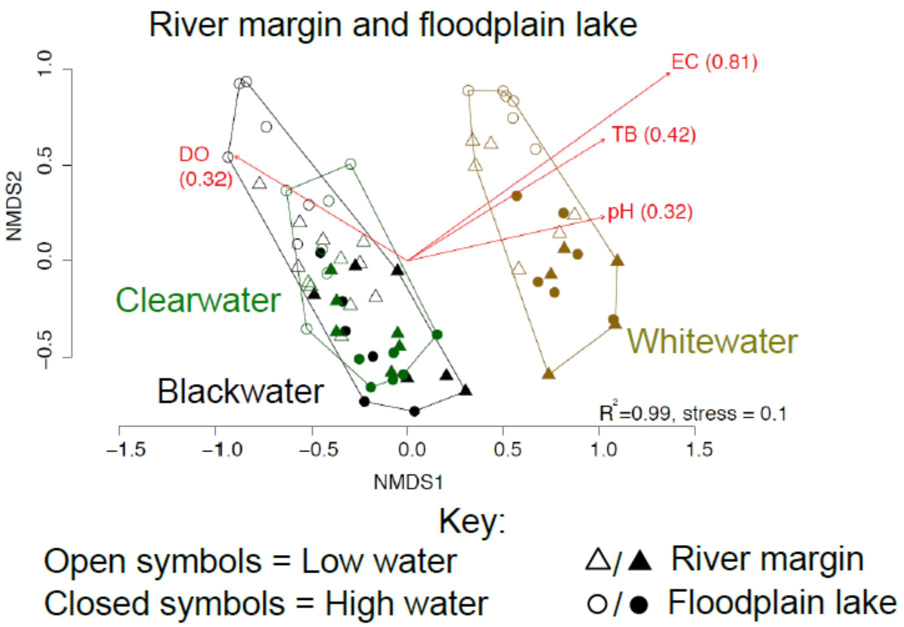

Fig. S2. Non-metric multidimensional scaling (NMDS) ordination based on fish assemblage structure. Here the analyses are divided by sampling gear into gill net and seine net. Polygons represent convex hulls for the three water types. Environmental vectors significant at $\alpha = 0.05$ are displayed with R^2 value in parentheses. DO = dissolved oxygen, EC = electrical conductivity, TB = turbidity, TC = temperature.

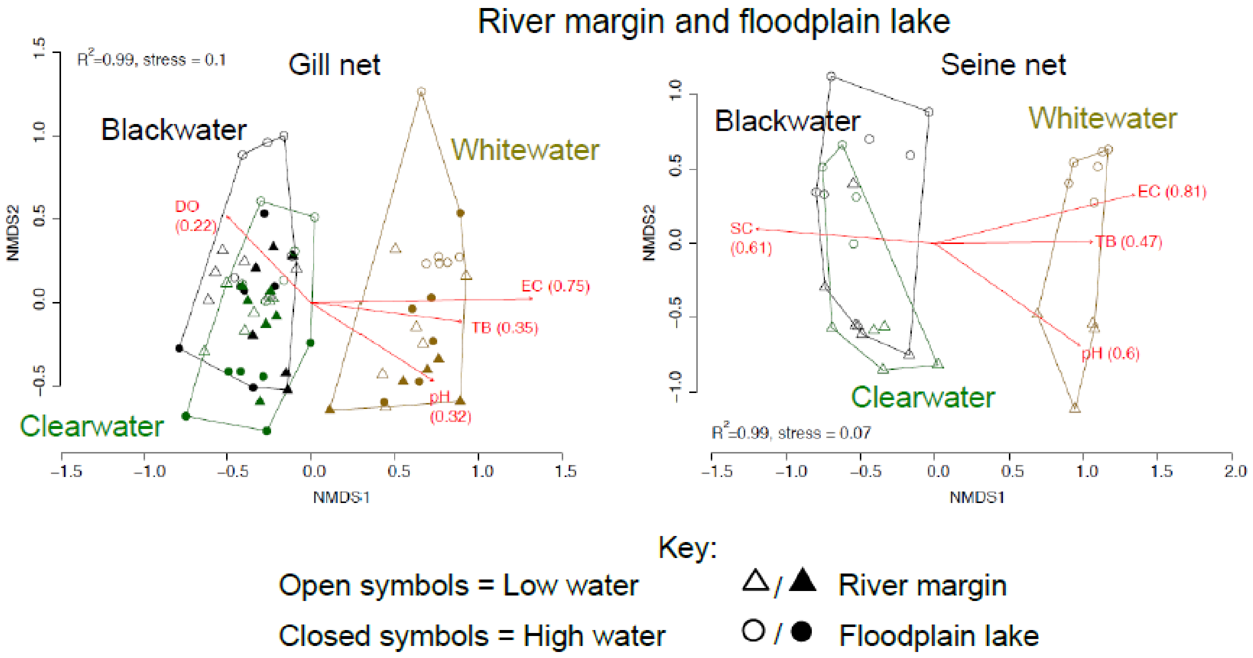

Fig. S3. Non-metric multidimensional scaling (NMDS) ordination based on fish assemblage structure. Here the analyses are divided by site replicate: site 1 (left column), and site 2 (right column). Polygons represent convex hulls for the three water types. Environmental vectors significant at $\alpha = 0.05$ are displayed with R^2 value in parentheses. DO = dissolved oxygen, EC = electrical conductivity, TB = turbidity, TC = temperature.

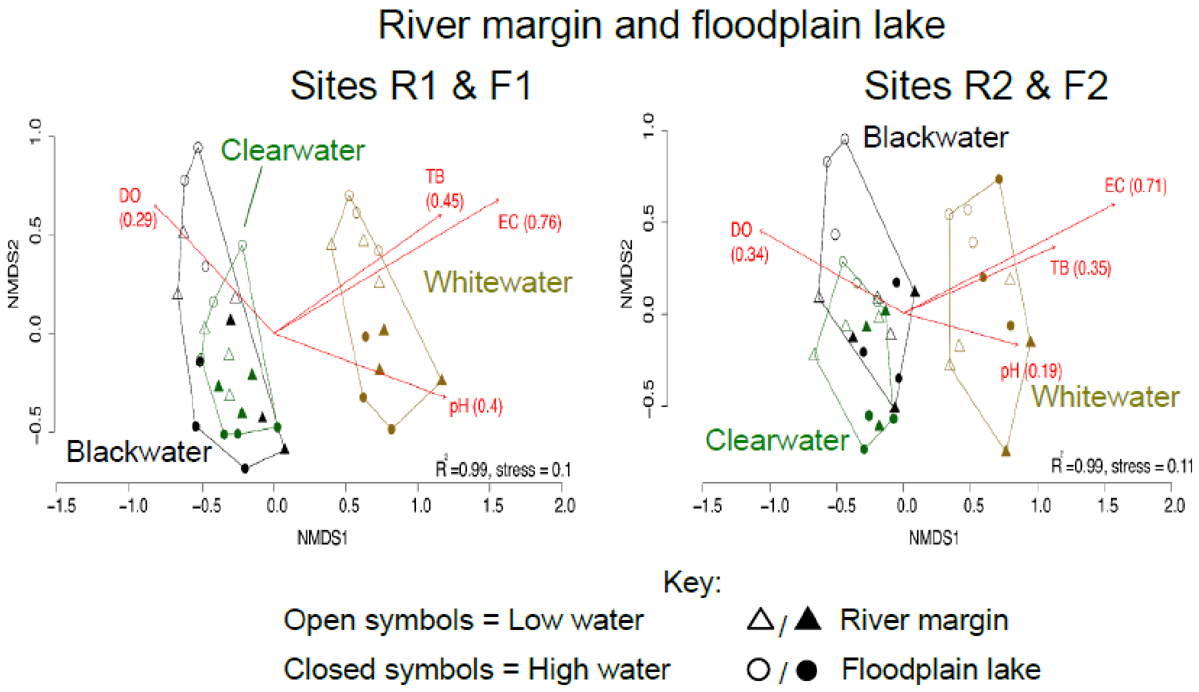

Fig. S4. Rarefaction curves for river margins (a) and floodplain lakes (b) divided by site replicate (1 = replicate sites R1 and F1, 2 = replicate sites R2 and F2, see Fig. 2 in main text).

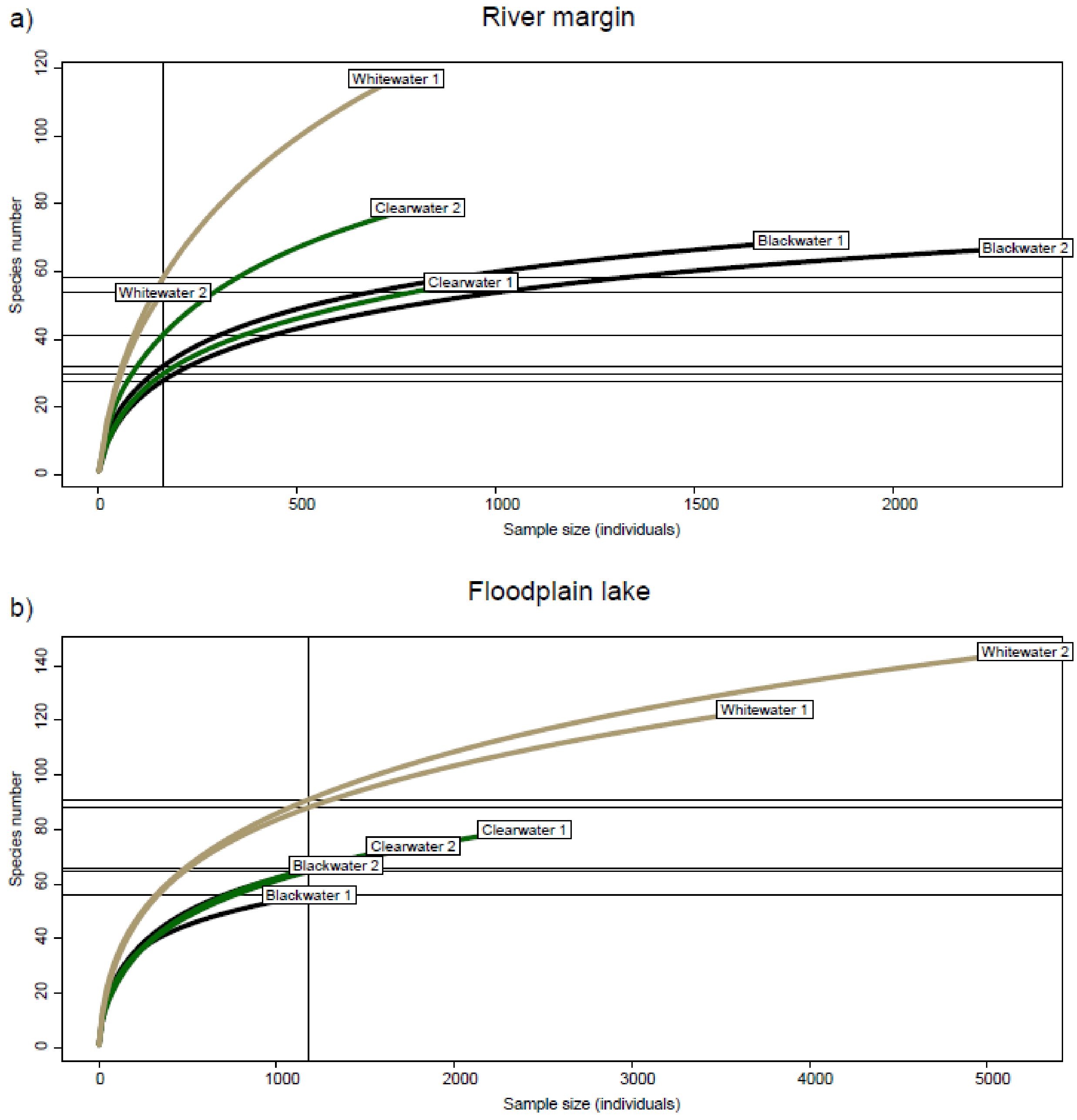

Fig. S5. Rarefaction curves for river margins (a) and floodplain lakes (b) divided by gear type (gill net; seine net) in the study area.

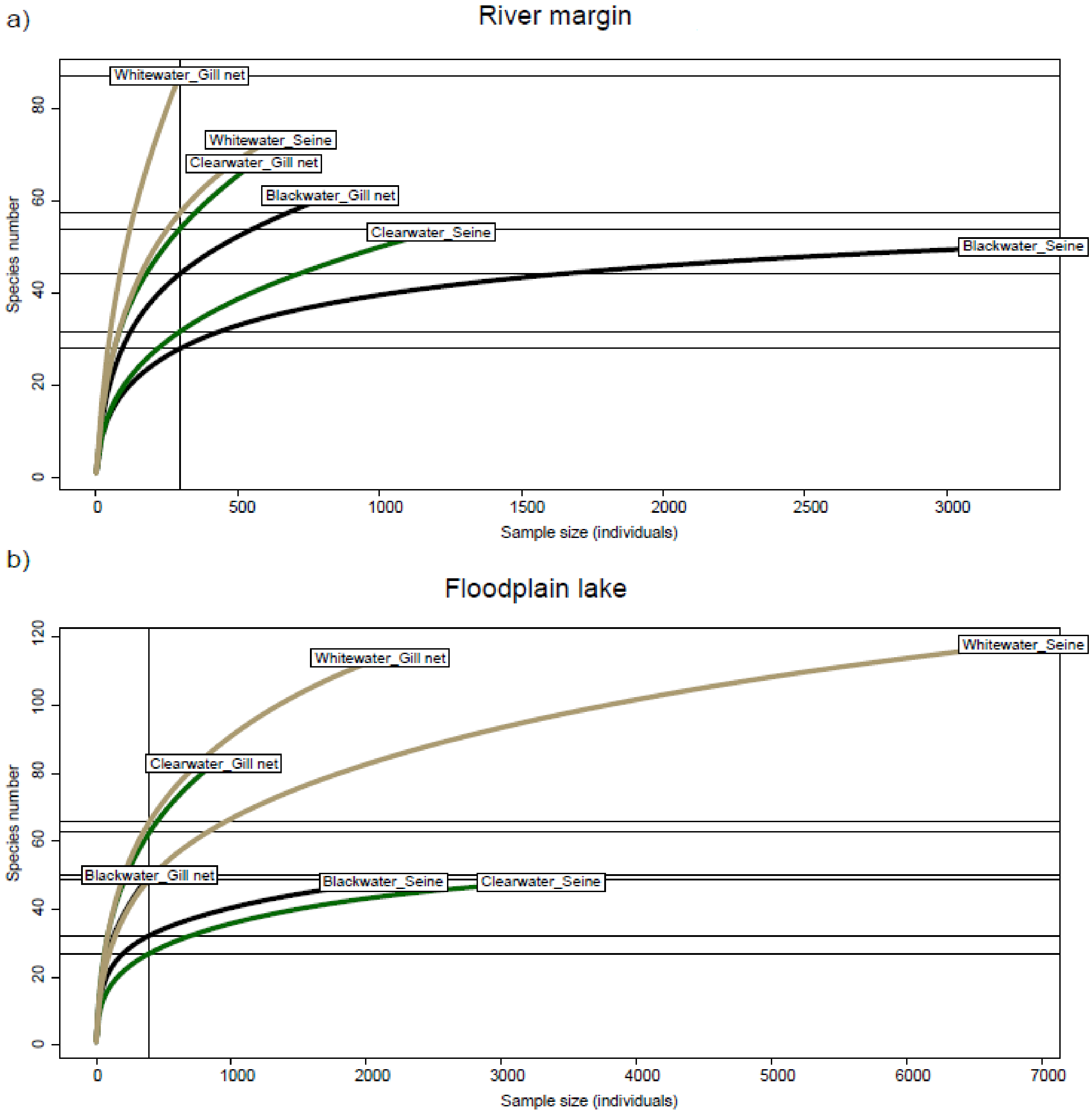

Fig. S6. Venn diagrams reporting species occurrences among the three Amazonian water types in the study area: BW = blackwater, CW = clearwater. WW = whitewater. Divided by Site replicates.

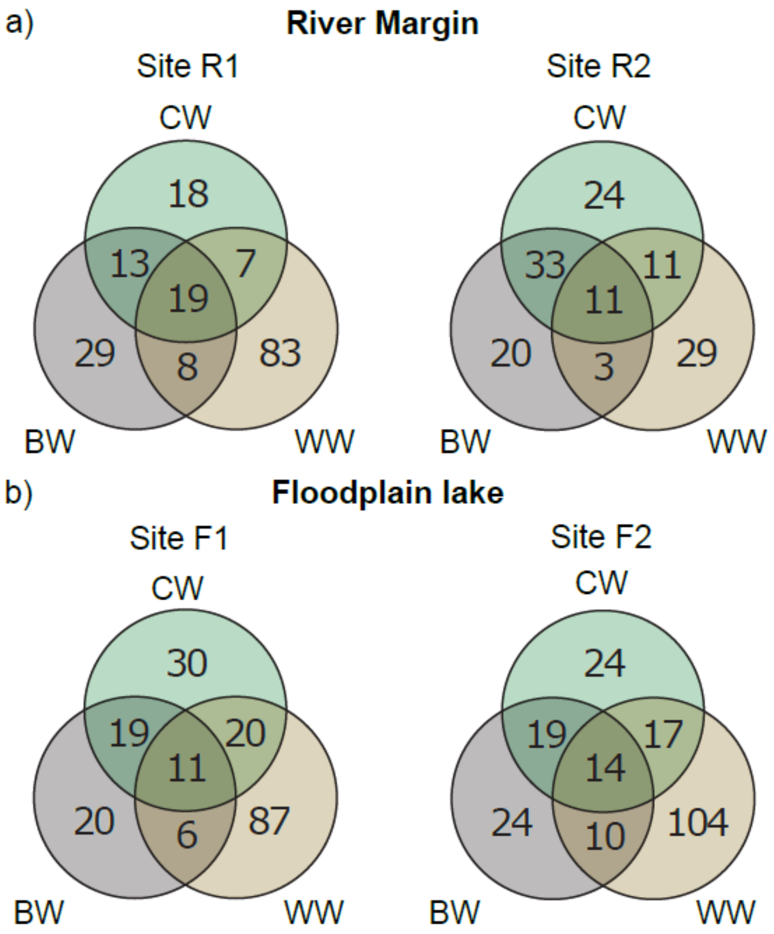

Fig. S7. Biomass (a), abundance (b) and species richness (c) for river margin and floodplain lake sites, summed across all 71 sampling events. Red crosses indicate outliers observations, defined as those above the 3rd quartile + [1.5 * interquartile range] or below the 1st quartile – [1.5 * interquartile range]).

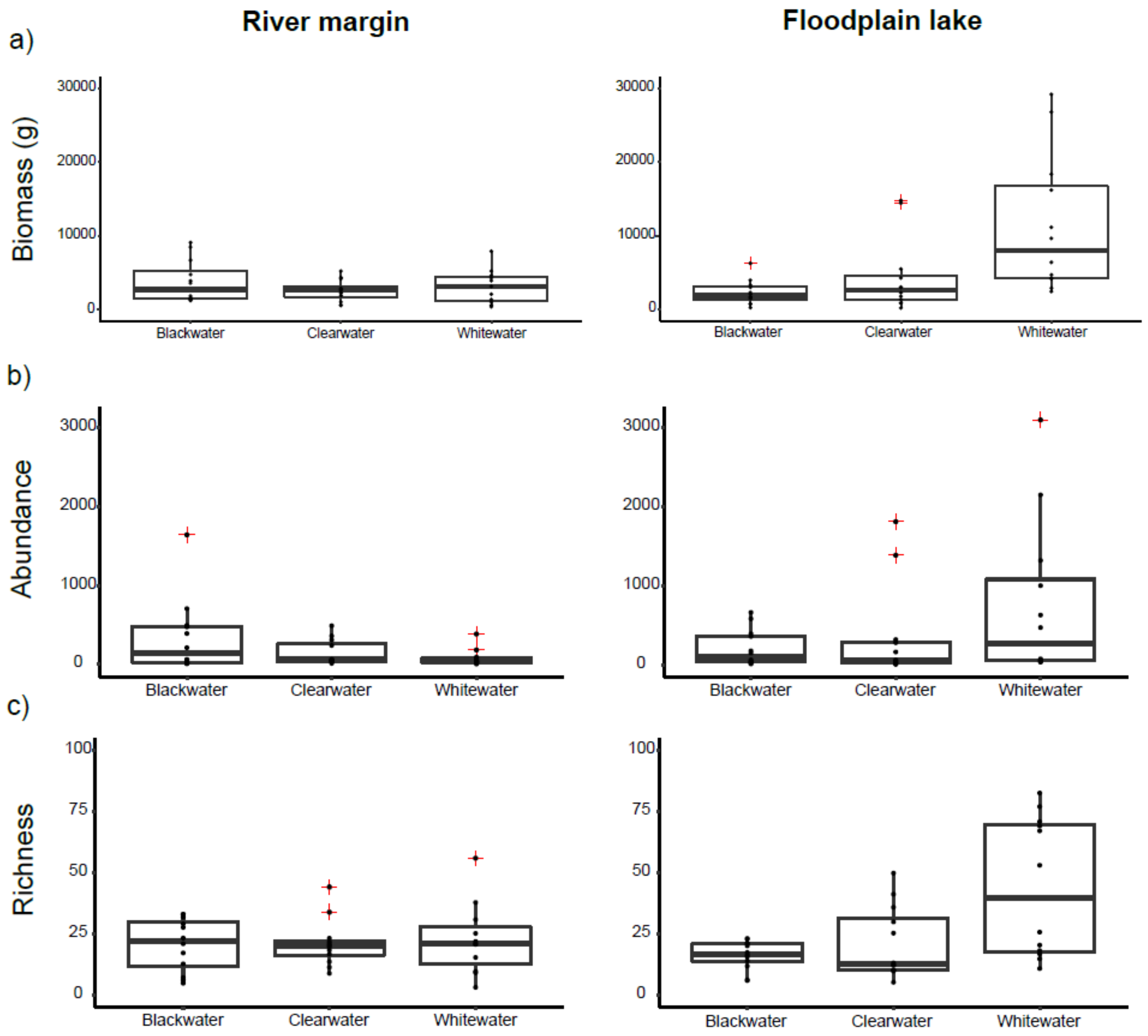

Supplement: Supplementary file 4 — Supplementary Figures. [file 41598_2020_72349_MOESM4_ESM.pdf]
